# Determinants of the Spatiotemporal Dynamics of the 2009 H1N1 Pandemic in Europe: Implications for Real-Time Modelling

**DOI:** 10.1371/journal.pcbi.1002205

**Published:** 2011-09-29

**Authors:** Stefano Merler, Marco Ajelli, Andrea Pugliese, Neil M. Ferguson

**Affiliations:** 1 Bruno Kessler Foundation, Trento Povo, Italy; 2 Mathematics Department, University of Trento, Trento Povo, Italy; 3 MRC Centre for Outbreak Analysis and Modelling, Department of Infectious Disease Epidemiology, Faculty of Medicine, Imperial College London, London, United Kingdom; Utrecht University, the Netherlands

## Abstract

Influenza pandemics in the last century were characterized by successive waves and differences in impact and timing between different regions, for reasons not clearly understood. The 2009 H1N1 pandemic showed rapid global spread, but with substantial heterogeneity in timing within each hemisphere. Even within Europe substantial variation was observed, with the UK being unique in experiencing a major first wave of transmission in early summer and all other countries having a single major epidemic in the autumn/winter, with a West to East pattern of spread. Here we show that a microsimulation model, parameterised using data about H1N1pdm collected by the beginning of June 2009, explains the occurrence of two waves in UK and a single wave in the rest of Europe as a consequence of timing of H1N1pdm spread, fluxes of travels from US and Mexico, and timing of school vacations. The model provides a description of pandemic spread through Europe, depending on intra-European mobility patterns and socio-demographic structure of the European populations, which is in broad agreement with observed timing of the pandemic in different countries. Attack rates are predicted to depend on the socio-demographic structure, with age dependent attack rates broadly agreeing with available serological data. Results suggest that the observed heterogeneity can be partly explained by the between country differences in Europe: marked differences in school calendars, mobility patterns and sociodemographic structures. Moreover, higher susceptibility of children to infection played a key role in determining the epidemiology of the 2009 pandemic. Our work shows that it would have been possible to obtain a broad-brush prediction of timing of the European pandemic well before the autumn of 2009, much more difficult to achieve with simpler models or pre-pandemic parameterisation. This supports the use of models accounting for the structure of complex modern societies for giving insight to policy makers.

## Author Summary

The 2009 H1N1pdm influenza pandemic spread rapidly but heterogeneously. A notable pattern occurred in Europe, with the UK exhibiting a first wave in early summer and a second wave in autumn, while all other European countries experienced a single wave in autumn/winter, resulting in a clear West to East pattern of spread. Our study asks which factors were most responsible for this variation, and to what extent the pattern of spread was predictable from data available in the first two months of the pandemic. Providing reliable answers to these questions would reduce uncertainty and improve situational awareness for policy-makers in the future, giving clearer expectations as to the likely impact and timing of a future pandemic and the potential effectiveness of mitigation measures. We found that that heterogeneity seen in 2009 can largely be explained by marked differences in school calendars, human mobility and demography across Europe. We also conclude that much of the variation in timing of the pandemic in Europe would have been predictable on the basis of data available in early June 2009. Our work supports the use of models accounting for the structure of complex modern societies for giving insight to policy makers in future pandemics.

## Introduction

In March 2009 H1N1pdm influenza emerged in Mexico and started spreading across the globe. Despite the rapidity in which the virus has reached a large number of countries in the world [1], transmission initially only became sustained in a subset of those countries seeded with infection from Mexico, notably the US and Southern hemisphere temperate countries. A relevant heterogeneity in the pattern of pandemic spread has been seen also within Europe: in that region, the UK has experienced a substantial first wave of transmission in the early summer, followed by a second one in the autumn, while all other European countries had only a limited transmission before the summer and a single wave in the autumn/winter [2]–[5]. Moreover, a clear West to East pattern of spread was observed for the 2009 pandemic [6], similar to that sometimes seen for seasonal flu [7].

Climatic differences (especially between northern and southern hemispheres) may be partly responsible for spatial heterogeneity in epidemic progression [8]. Human mobility patterns can also affect the spatiotemporal dynamics of an epidemic [9], [10] as well as heterogeneity in the population itself - sociodemographic structure can affect the susceptibility and contact patterns [10], [11]. For the 2009 H1N1 pandemic, the timing and length of summer school holidays [12], [13], given the emergence time, may have also affected the timing of pandemic spread in Europe.

By employing an individual-based stochastic simulation model, structurally similar to those already developed for predicting the spatiotemporal spread of a flu pandemic in different geographic areas [10], [14]–[22], we analyse here which factors are most responsible for the observed geographical differences, and to which extent the pattern was predictable on the basis of the first available data on the spread of H1N1pdm in Mexico [23], the US and the UK [24]. Thus, we do not fit the model to the observed pattern of spread (which is possible only after the pandemic); rather, we use parameter values estimated from the first published analyses and examine the extent to which the model predicted spread agrees with the pattern of spread seen in the Europe in the summer and autumn of 2009. We employ extensive sensitivity analysis to assess the uncertainty in prediction, as well as the extent to which the predictions could have been improved by better parameterisation or greater detail. This allows us to also evaluate the predictability of the patterns seen and to discuss implications for the control of future pandemics.

## Methods

Our analysis makes use of an individual-based stochastic simulation model structurally similar to a model previously developed for Europe [10]. The simulation is a spatially-explicit discrete-time SEIR model with force of infection decreasing with the geographical distance which explicitly models transmission in households, schools and workplaces. Country-specific sociodemographic data from Eurostat [25] were used to parameterise the distribution of individuals in households, schools and workplaces. Infection spread between countries is modelled through cross-border diffusion and long-distance travel, making use of European air and railway transportation data.

Previous work using this model [10] did not specifically aim to model the 2009 H1N1 pandemic, but rather examined the impact of human mobility patterns and demographic heterogeneity on the expected spread of a ‘generic’ influenza pandemic, parameterised to reproduce the transmissibility of the pandemics seen in the last century. The version of the model used in that work [10] lacked some key features required to realistically capture the epidemiology of the 2009 pandemic.

For this paper, we enhanced the simulation framework in a number of ways. First, rather than modelling the importation of cases through a simple compartmental model describing the spread of a pandemic outside the EU, here we explicitly model importation of cases from Mexico and US into EU countries. Since it is apparent that many features of pandemic spread in Europe depend on the timing of its emergence, the simulation in this study is synchronized to match the time of the first recorded cases across Europe. Specifically, the epidemic is seeded using country-specific data on travel-related cases in the early phase of the epidemic (up to June 3, 2009) [26]. Second, instead of assuming adults and children are equally susceptible to infection [10], [14]–[20], here we model children as being twice as susceptible to infection as adults, based on early analyses of the pandemic in Mexico [23] and the UK [24]. Third, the key role children played in the transmission of the 2009 pandemic meant that we incorporated the timing of school holidays in different EU countries, and the impact of those holidays on transmission. Fourth, the model of long-distance travel was refined to take account of the duration of stays abroad in estimating transmission risk between travellers and host populations. Last, the parameterisation of transmission rates in households, schools and workplaces was refined to match available data [12], [17], [27].

Beyond the socio-demographic information used [10], we parameterised the model using information available up to June 2009 on the generation time of the pandemic virus, *T*_g_, and on the value of the reproduction number, *R*_0_ [23], [24]. Overall, the model has five transmission parameters: the transmission rate in households, in schools, in workplaces, in the general community and during long-distance travel. These are assumed to be identical for all European countries. For a given choice of *T*_g_, once the transmission parameters are fixed, one can estimate a value of *R*_0_ for the model from the growth rate of the simulated epidemic. *R*_0_ will differ between countries because of the sociodemographic differences even keeping transmission parameters constant; our reference value for comparison with data is that obtained from simulations of the pandemic in the UK, *R*_0_^UK^.

We assigned the value of the five transmission parameters in such a way that *R*_0_^UK^ matched early estimates of *R*_0_ from UK data (achieved by applying an overall scaling to all transmission coefficients), and so that the proportion of transmission in different social contexts matched that estimated in past work [12], [17]: after adding the effect of age-dependent susceptibility, this results in 36% of cases being transmitted in schools, 31% in households, 9% in workplaces and 24% in the general community. During school holidays, no transmission is assumed to occur in schools, while community transmission is increased by a factor of 1.4 [12], to account for increased non-school contacts among students. The model predicts, using data on the number of nights spent by European citizens in EU countries outside their own member state, that the percentage of infections during long-distance travel is slightly lower than 0.5%.

By the end of June 2009, the most reliable estimates of epidemic growth rate for the H1N1pdm pandemic were those obtained from the comprehensive (>25% population coverage) Qsurveillance sentinel surveillance system for influenza-like-illness operating in England [28]. Fitting an exponential model with non-zero intercept to data from the Qsurveillance network data available to July 1 [29] the estimated real-time exponential growth rate is 0.141/day (95% CI: 0.127–0.156), corresponding to a doubling time of 4.9 days (95% CI: 4.4–5.5 days). Such epidemic growth rates can be translated into estimates of the reproduction number, *R*_0_, given estimates of the generation time distribution [30]. For instance, assuming exponentially distributed latent and infectious periods (as the simulation model used here does) with means of 1.5 days and 1.6 days respectively, the corresponding reproduction number estimate is 1.48 (95% CI: 1.43–1.54). Similar estimates (see supporting Text S1) are obtained using the H1N1pdm case estimates generated by the UK Health Protection Agency (HPA) which are derived from ILI data weighted by the proportion of ILI cases each week testing positive for H1N1pdm via virological surveillance, albeit the confidence bounds are wider due to the relatively small numbers of samples which were virologically tested.

We therefore choose to illustrate the qualitative predictions of the model with default values of *R*_0_ = 1.48 and *T*_g_ = 3.1 days (parameters in Table 1). This choice of generation time is a little longer than the modal estimate derived from (mostly household based) contact tracing data in the early UK epidemic [24], but lies between lower and higher past estimates of the generation time of influenza [15]–[17], [31]. A sensitivity analysis is presented which demonstrates relative insensitivity of the ability of the model to predict timing of the epidemic across Europe to variation in *R*_0_ and *T*_g_, so long as the epidemic doubling time observed in the UK in June is reproduced.

**Table 1:** Epidemiological parameters used in the baseline simulations (*R_0_* = 1.48, *T_g_* = 3.1 days).

Parameter	Value
Transmission rate in households	0.711 days^−1^
Transmission rate in schools	0.840 days^−1^
Transmission rate in workplaces	0.408 days^−1^
Transmission rate in the general community	0.319 days^−1^
Transmission rate during long-distance travel	4.252×10^−15^ days^−1^
Transmission rate for modelling importation of cases	0.832 days^−1^
Latent period	1.5 days
Infectious period	1.6 days
Relative susceptibility to infection of adults with respect to children	0.5

Details on the model structure and its parameterisation are given in the supporting Text S1.

## Results

Most trips from the US and Mexico to the EU are to Western European countries and especially to the UK (about one third of the trips, see supporting Text S1). We found that the date of the first case in each European country [32] correlates significantly with the number of travellers to the country from Mexico and US, both in the data and in model output (see Table 2) and with longitude (a West to East pattern is observed; see Table 2).

**Table 2:** Correlation of population variables and epidemic statistics as observed or predicted by the model in the different European countries.

Epidemic statistic	Population variable	Correlation as observed in the data		Correlation as predicted by the model	
Day of the first case^h^	US-MX travellers a	ρ = −0.875	p<0.001	ρ = −0.807	P<0.001
Day of the first case^h^	Longitude b	ρ = 0.618	p<0.001	ρ = 0.373	p = 0.02
Day of the first case^h^	GDP c	ρ = −0.548	p = 0.002	ρ = −0.255	p = 0.12
Peak week^i^	Inter-EU passengers d	ρ = −0.519	p = 0.01	ρ = −0.685	p<0.001
Peak week^i^	Longitude b	ρ = 0.584	p = 0.002	ρ = 0.743	p<0.001
Peak week^i^	GDP c	ρ = −0.585	p = 0.002	ρ = −0.745	p<0.001
Cumulative attack rate	Household size e			ρ = 0.702	p<0.001
Cumulative attack rate	Average age f			ρ = −0.861	p<0.001
Cumulative attack rate	Fraction of children g			ρ = 0.785	p<0.001
Peak weekly attack rate	Household size e			ρ = 0.734	p<0.001
Peak weekly attack rate	Average age f			ρ = −0.863	p<0.001
Peak weekly attack rate	Fraction of children g			ρ = 0.725	p<0.001

a US-MX travellers: the yearly number of travellers entering the country from US and Mexico as resulting from the analysis of air travel data [25].

b Longitude: the longitude of the capital city of the country.

c GDP: per capita gross domestic product of the country [25].

d Inter-EU passengers: the yearly number of travellers entering the country from other European countries as resulting from the analysis of air travel data [25].

e Household size: the average number of members of households in the country [25].

f Average age: the average age (in years) of the population of the country [25].

g Fraction of children: the fraction of individuals aged less than 15 years old in the population of the country [25].

h Day of the first case: obtained from the analysis of the WHO daily situation updates for pandemic (H1N1) 2009 [32].

i Peak week: obtained from the analysis of the WHO situation update from week 40/2009 to week 07/2010 [6].

**Figure 1:**
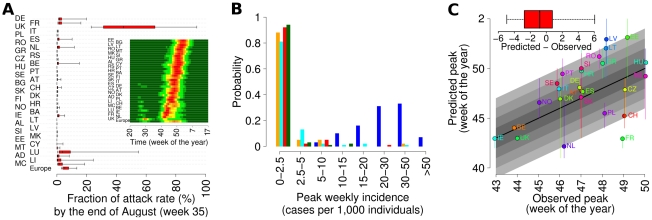
Timing of the pandemic (*R_0_* ^UK^ = 1.48 *T_g_ = *3.1 days). (A) Distribution of the fraction of predicted attack rate (2.5%, 25%, 50%, 75% and 97.5% percentiles) by the end of August (week 35) in the different countries. In the inset, mean incidence per week in the different European countries in colour scale, from dark green (less than 5 per 1,000) to dark red (above 50 per 1,000). (B) Probability of observing a summer wave with peak incidence in a given range, in UK (blue), Germany (cyan), Netherlands (orange), Ireland (green) and Spain (red). (C) Observed peak week plotted versus predicted peak week (vertical bars represent 95% confidence intervals of the predictions) for European countries covered by the WHO/Europe weekly influenza surveillance system; only the autumn wave is considered for UK. In the inset minimum, 25%, 50%, 75% percentiles and maximum of observed minus predicted peak week. A total of 100 simulations were undertaken to produce the results shown.

The overall pattern of infection spread is summarised in Fig. 1A, where the distribution (as predicted by the model) of the proportion of expected cases before the end of the summer among the total case is shown for each country, together with the predicted infection level (average of all simulations) for each country and each week. From the picture, one sees that a sizeable proportion of all cases was to be expected before the end of the summer in UK (the predicted value is above 20% in all simulations). As for other countries, one sees that in the large majority of simulations only a small proportion of cases would have been expected before the end of the summer (<3% in 75% of simulations), while in few simulations a more substantial summer wave (due to the rapid build-up of cases) occurs in a few countries (mainly Germany, France, Netherlands and Spain). As it is shown in the inset of Fig. 1A, incidence rates in early summer would only have been expected to be high in the UK. Fig. 1B shows the distribution of predicted spring-summer peak incidence from multiple runs of the model for a few selected countries; again it is seen that a substantial summer wave in UK is predicted as almost certain, while only a low probability of a minor peak is predicted for the other countries.

The analysis of the first 711 laboratory-confirmed cases of H1N1pdm influenza in Europe available at June 3 indicated that 452 (64%) were probably infected overseas [26]. However, a key part of the protocol adopted in most European countries for testing people for H1N1 infection was travel links to known affected countries. Therefore it is perhaps unsurprising that a high fraction of reported confirmed cases by July were in travellers. What has previously been unknown is the number of undetected cases occurring in the early stages of the European pandemic. Comparing the proportion of reported confirmed cases which were imported with our model predictions, we estimate that 8% (95%CI: 1%–22%) of indigenously transmitted cases were being detected in Europe up to June 3^rd^.

Fig. 1C compares the model-predicted week of autumn peak incidence with that observed from ILI surveillance data for the countries for which data was available from the World Health Organisation (WHO) [6]. The correspondence between predicted and observed appears quite good for almost all countries (Spearman's correlation coefficient between data and predictions 0.53, p = 0.006), considering that no data fitting has been employed. From the subpanel in Fig. 1C, one can see that the error is below 2.5 weeks for 50% of countries, while the average error is computed to be around 2.2 weeks. The autumn peak week in each European country correlates significantly with the number of travellers to the country from other European countries, both in the data and in model output (see Table 2). Significant correlations with longitude (a West to East pattern is observed) and gross domestic product are also seen (Table 2).

Model predictions aim at tracking the epidemic trajectory (and not only peak timing) and estimating the community burden of infection over time. One (albeit imperfect) measure of infection rates is provided by ILI data; however it should be noted that as this only measures those seeking healthcare, ILI incidence represents a (largely unknown) fraction of true infection incidence. In addition, ILI is a non-specific measure, as multiple other pathogens can cause ILI-like symptoms. We therefore focus on comparing incidence rates at different times in the pandemic, rather than attempting to model the absolute magnitude of ILI incidence. UK data is of particular relevance therefore, due to the availability of serological data [33], [34] that allow for some absolute quantification of infection rates.

**Figure 2:**
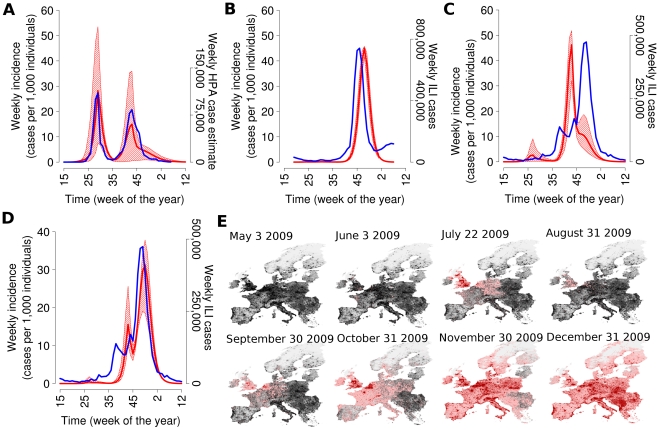
Spatiotemporal spread of the European pandemic (*R_0_* ^UK^ = 1.48 *T_g_* = 3.1 days). (A) Comparison between average weekly incidence in UK as predicted by the model (red) and weekly HPA case estimates (blue). Red shaded area represents 95% confidence intervals of the expected weekly incidence over time. (B) Comparison between average weekly incidence in Italy as predicted by the model (red) and weekly ILI cases [35] (blue). (C) Comparison between average weekly incidence in France as predicted by the model (red) and weekly ILI cases [37] (blue). (D) As (C) but assuming *R_0_* ^UK^ = 1.43. (E) Time sequence (in days) of a single simulation with the first European case in UK is shown. Colours from pink to dark red indicate an increasing number of daily cases (dark red indicates more than 10,000 daily cases). A total of 100 simulations were undertaken to produce the results shown.

Simulations for UK (see Fig. 2A) show two waves of comparable size, roughly in agreement with the profile of observed ILI incidence, though recently available serological data [34] suggest that the autumn wave was considerably larger than the summer wave; from Fig. 2A one can see that a summer wave was almost certain (credible interval for peak week incidence between 8 and 53 per 1,000) while the peak of the autumn wave could be all the way between minimal and 35 per 1,000; depending on the simulation, either wave could be the largest: it is clear that the prior prediction of the magnitude of both waves would always have been challenging. The timing of the peaks of the two waves is strongly determined by the dates of summer and autumn school holidays.

Simulations for Italy (see Fig. 2B), a country with only one clear wave in autumn, are similar to observed ILIs [35], except for a 2 week delay (virological data [36] suggest that the increase in incidence in January 2010 was due to B-type viruses). Finally, we show simulations for France (see Fig. 2C), the country with the largest difference (6 weeks) between predicted and observed peak week (Fig. 1C); simulations show a big peak at week 43 followed by a sudden drop due to school holidays in weeks 44 and 45 with a possible minor increase after school re-opening, while data show a major increase in ILIs only after school re-opening [37]. However Fig. 2D shows that small variations in the assumed value of *R*_o_ can cause large differences in the predicted peak week for France, due to the timing and unusual length (2 weeks) of autumn school holidays in that country.

A typical European pandemic simulation is shown in Fig. 2E: the epidemic develops early in the UK, because of the large number of travellers and the late date of school closure for summer holidays in England and Wales. When schools close in England and Wales the incidence there decreases sharply, and remains very low in all European countries until the autumn when an epidemic wave occurs in all countries with a general West to East trend depending on mobility patterns and economic factors (Table 2). Such a trend has been observed for seasonal flu [7] as well as the 2009 pandemic [6].

**Figure 3:**
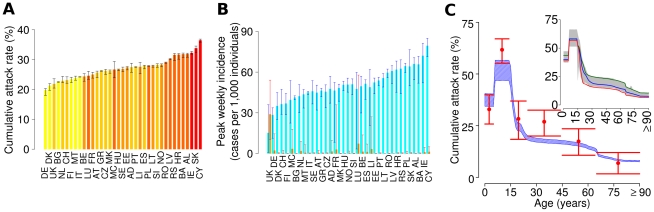
Variation in attack rates by age and country (*R_0_* ^UK^ = 1.48 *T_g_* = 3.1 days). (A) Average cumulative attack rate predicted by the model in the different European countries (black bars represent 2.5% and 97.5% percentiles of the distribution). Colours represent the fraction of individuals <15 year old in the population, increasing from yellow (13%) to red (22%). (B) Average peak weekly incidences predicted by the model in the different countries for the summer (orange) and the autumn (cyan) waves; for each country and wave, 2.5% and 97.5% percentiles of the distribution are shown (red and blue bars). (C) Post pandemic age-stratified attack rates. Estimates of post pandemic seroconvertion rates in England [34] (precisely, differences between the percentage of post pandemic (2010) serum samples from England with HI 1∶32 or more, and corresponding percentages in serum samples obtained in 2008 in England) against cumulative attack rates by age in UK predicted by the model at the end of the pandemic: red points represent the expected value of post pandemic seroconversion rates (vertical lines represent 95% confidence intervals), shaded blue areas represent 95% confidence intervals of model simulations. Cumulative attack rates by age as predicted by the model at the end of epidemic in different European countries are shown in the inset: shaded grey area represent 95% confidence interval at European level, while solid lines represent the median for Italy (blue), Germany (red) and Ireland (green). A total of 100 simulations were undertaken to produce the results shown.

The model yields also quantitative predictions about cumulative infection attack rate, peak incidence, and the age distribution of cases for all European countries. Most interestingly, the model predicts substantial variation in cumulative attack rate across Europe, with values from 19.8% (95% CI 18–20.3) in Germany to 36.4% (95% CI 35.8–36.9) in Cyprus (see Fig. 3A) with an average (over Europe) of 24.8% (95% CI 24.1–25.3). The standard deviations around these estimates are small, except for the countries with small populations or a summer wave.

Recently available data [34] on antibody prevalence in England allow for a comparison with model predictions for the UK (see Fig. 3C); except for the age group 25–44, where the model slightly underestimates the number of cases, the agreement is excellent, considering that no parameter fitting has been performed. However, it should be noted that this comparison could be partly affected by the confounding effects of vaccination – while UK vaccination rates were low overall, the samples tested for these serological studies were from hospital patients who may have had a higher vaccination rate. Nonetheless, UK data are the only data available to date allowing for such comparison. Further such comparisons will be possible, once serological data collected from other countries become available.

The predicted peak weekly incidence in the autumn ranged from 1.5% (95% CI 0.1–3.6) in UK to 7.9% (95% CI 7.1–8.5) in Cyprus (see Fig. 3B). The final size of the epidemic (*i.e.* the cumulative infection attack rate) is strongly affected by demographic differences: cumulative attack rate is positively correlated with the fraction of individuals aged less than 15 years (see Fig. 3A and Table 2) and average household size (see Table 2). Note that while predictions of the overall population attack rate are very different among countries, age-specific attack rates are much more uniform (see Fig. 3C).

### Sensitivity analyses

An extensive analysis of the sensitivity of these results to model assumptions is presented in the supporting Text S1; here we summarise some results concerning the assumed reproduction number *R*_0_ and generation time *T*_g_, age-depending susceptibility, inter-European mobility, demographic heterogeneities, school calendar and seeding of infection into Europe.

**Figure 4:**
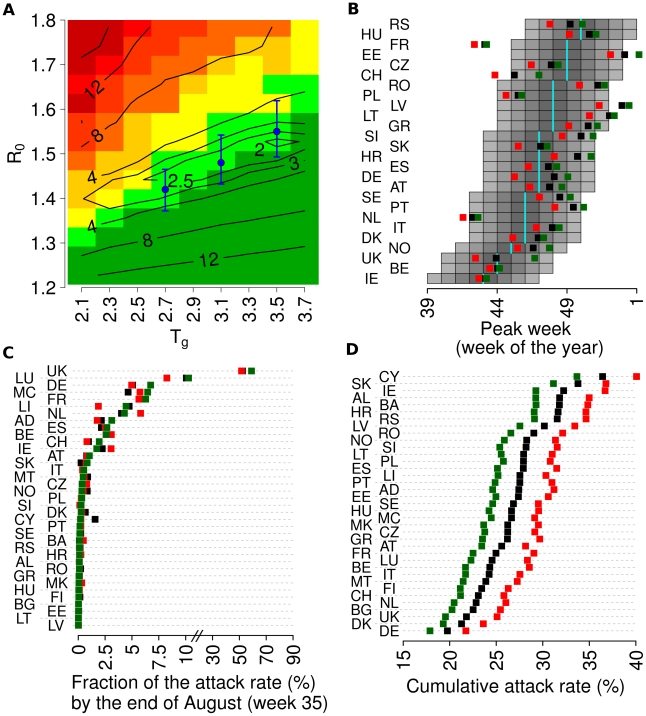
Sensitivity to assumed *R*_0_ and *T*_g_. (A) Sensitivity analysis by varying *R_0_* ^UK^ and *T_g_*: level curves (and numbers) in black represent the mean deviation between observed and predicted peek week (in weeks). Colours represent the expected number N of countries with peak of the summer wave above 30 per 1000 individuals, ranging from dark green (N = 0) to dark red (N>30). Light green indicates 0.5<N< = 1.5, yellow indicates 1.5<N< = 2.5 and light orange indicate 2.5<N< = 5. Blue points represent possible pairs (*R_0_* ^UK^, *T_g_*) as resulting from the Qsurveillance data: *R_0_* ^UK^ = 1.42, *T_g_* = 2.7 days; *R_0_* ^UK^ = 1.48, *T_g_* = 3.1 days; *R_0_* ^UK^ = 1.55, *T_g_* = 3.5 days. Blue vertical lines represent the uncertainty of *R_0_* ^UK^ as resulting from the uncertainty of the growth rate *r* of the Qsurveillance data. (B) Peak week for European countries covered by the WHO/Europe weekly influenza surveillance system as observed (cyan bars), as predicted by simulations with *R_0_* ^UK^ = 1.48 and *T_g_* = 3.1 days (black squares), as predicted by simulations with *R_0_* ^UK^ = 1.42 and *T_g_* = 2.7 days (green squares) and as predicted by simulations with *R_0_* ^UK^ = 1.55 and *T_g_* = 3.5 days (red squares). (C) As (B) but for the fraction of the attack rate by end of August. (D) As (B) but for the cumulative attack rate. A total of 100 simulations were undertaken for each parameter set to produce the results shown.

Fig. 4A shows how the average deviation between predicted and observed peak week depends on *R*_0_^UK^ and *T*_g_. The three blue points in the figure represent combinations of these parameters which are compatible with the observed doubling time of the UK summer epidemic (see Methods), namely *R*_0_^UK^ = 1.42, *T*_g_ = 2.7 days, *R*_0_^UK^ = 1.48, *T*_g_ = 3.1 days (the baseline values) and *R*_0_^UK^ = 1.55, *T*_g_ = 3.5 days. It can be seen that they all lie in a narrow strip leading to satisfactory predictions, both in terms of the timing of autumn epidemic peak, and in the predicted presence of a UK summer wave. In fact, for parameter values which yield an adequate prediction of autumn peak week, the expected number of countries with a summer wave above threshold is approximately 1, *i.e.* the UK. Thus, model predictions are not very sensitive to the values of *R*_0_^UK^ and *T*_g_ so long as we consider simulations consistent with the doubling time observed in the initial phase of the epidemic in UK.

Fig. 4B shows that (as expected) simulations with *R*_0_^UK^ = 1.42 and *T*_g_ = 2.7 days and with *R*_0_^UK^ = 1.55 and *T*_g_ = 2.5 days give rise to slightly (approximately 1 week) slower or faster simulated epidemics in autumn respectively with respect to the baseline simulations. There are no substantial differences in the predicted scale of summer spread between these parameter sets (Fig. 4C), while, as expected, substantial differences emerge in terms of cumulative attack rate by the end of the pandemic (Fig. 4D).

Fig. 4A also shows that values of *R*_0_^UK^ and *T*_g_ in the right bottom corner (low *R*_0_^UK^ and long *T*_g_), a summer wave would not be expected in any country and the epidemic would have spread much more slowly, thus delaying the epidemic peaks in autumn by months; while with high *R*_0_^UK^ and short *T*_g_, several countries would have experienced a summer wave and epidemics would have peaked earlier across Europe. Thus, the observed pattern of spread might have been dramatically different, with much earlier peaking of transmission, had the transmissibility of H1N1 been comparable to that seen in previous pandemics such as 1957 and 1968.

**Figure 5:**
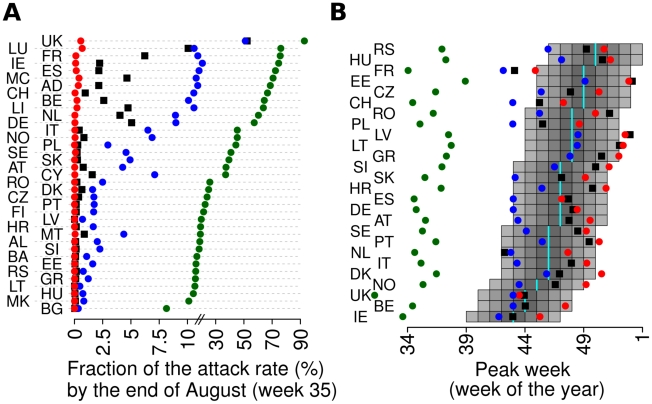
Effects of school holidays (*R_0_* ^UK^ = 1.48 *T_g_* = 3.1 days). (A) Fraction of all infections expected by end of August (week 35) as predicted by baseline simulations (actual school calendars in all countries, including autumn holidays, black squares), by assuming no holidays (green circles), by assuming the school calendar of Finland in all countries (schools close on 30 May, red circles) and by assuming the school calendar of UK in all countries (schools close on 20 July, blue circles). (B) Peak week for European countries covered by the WHO/Europe weekly influenza surveillance system as observed (cyan bars), as predicted by baseline simulations (black squares), by assuming no holidays (green circles), by assuming the school calendar of Finland in all countries (red circles) and by assuming the school calendar of UK in all countries (blue circles). A total of 100 simulations for each parameter set were undertaken to produce the results shown.

The effect of the timing of school holidays on the magnitude of a summer wave also proved to be substantial, as shown by simulations where the same school calendar was given to all countries, causing (according to the calendar chosen) a different pattern of summer waves (Fig. 5A), and a lack of correlation between predicted and observed week of peak incidence in the autumn (Fig. 5B). By assuming the school calendar of Finland in all European countries, it emerges that no country would have experienced any summer wave. Moreover, a slight delay in the autumn peak week would have been observed (especially in countries less connected with US and Mexico) due to the lack of a well established epidemic in the United Kingdom and other countries in the Western part of Europe during the summer. The opposite pattern is observed by assuming the school calendar of the United Kingdom in all European countries. A summer wave would have been likely in the great majority of countries (especially in the Western part of Europe) and an anticipation of the autumn peak week would have been observed, due to epidemics ongoing in many Western countries and triggering the start of epidemics in the Eastern part of Europe. Without school holidays, a relevant single summer wave would have been observed in almost all European countries. These results highlight the role of school holidays in determining the epidemic timing, although an additional impact of climatic factors cannot be ruled out.

**Figure 6:**
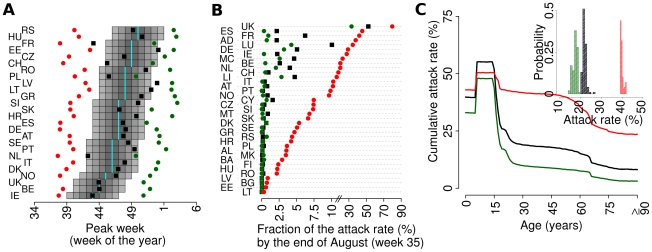
Effects of age-dependent susceptibility to infection (*R_0_* ^UK^ = 1.48 *T_g_* = 3.1 days). (A) Peak week for European countries covered by the WHO/Europe weekly influenza surveillance system as observed (cyan bars), as predicted by baseline simulations (susceptibility of children 2 fold greater than that of adults, black), by assuming that susceptibility of children and adults was identical (red) and by assuming children were 4-fold more susceptible than adults (green). (B) As (A) but showing the fraction of the attack rate by end of August. (C) As (A) but showing mean cumulative attack rates by age in UK. The inset shows the distribution across simulations of the cumulative attack rates in UK in the three scenarios. A total of 100 simulations for each parameter set were undertaken to produce the results shown.

By assuming no difference in susceptibility between adults and children (but still calibrating the model on the epidemic growth rates seen in the UK summer wave), the model predicts much faster epidemics in autumn (Fig. 6A), because of the lower impact on transmission of the closure of schools, with sizeable summer waves in several countries (Fig. 6B). In addition, one would have expected a much larger attack rate in adults and the elderly (Fig. 6C). Increasing the difference in susceptibility to a factor of 4 between adults and children causes much lower attack rates in adults (Fig. 6C) but also some delay in timing of peaks (Fig. 6A). These results highlight how different the pattern of epidemic spread might have been had adults been more susceptible to the 2009 virus. They also suggest that the susceptibility to infection of children relative to adults is likely to have been closer to 2 than 4, but that overall, capturing age differences in susceptibility to infection (a feature not present in pandemic models developed before the H1N1 pandemic) is necessary to capture the observed pattern of spatiotemporal spread of H1N1pdm in Europe. This highlights the need to obtain early estimates of differential susceptibility with age in any future pandemic.

We also examined a model variant which assumes that all countries have the same socio-demographic structure, thus neglecting inter-European heterogeneities (see supporting Text S1). The net effect on pandemic speed and attack rate is similar to increasing or decreasing (depending on the country's demography) *R*_0_. The attack rates become identical in almost all countries (slightly lower attack rates are estimated for countries which experience a sizeable summer wave, due to the mitigating effect of the summer closure of schools in reducing the final size of the epidemic [12], [38]), and the timing of the peak of the epidemic depends only on school calendar and importation of cases from US and Mexico, causing marked differences from the basic model, in which the demography also plays a role: for instance, if all countries had the same demographic structure as Germany, a summer wave would be unlikely in any country (including UK); if all countries were like Ireland, a sizeable summer wave would be likely in several countries (most noticeably Germany).

Finally, the assumptions we used in modelling the seeding of H1N1pdm infection into the EU was varied in several ways (see supporting Text S1). For instance, assuming that importation of cases is proportional to the number of travellers from US and Mexico from air travel data (and not to country specific data on observed imported cases, as in the baseline simulations), leads to overestimates of the number of imported cases for some countries with international airport hubs (e.g. the Netherlands and Germany) and to underestimates of imported cases for Spain. However, the overall pattern of summer waves and autumn peaks does not change substantially.

Further sensitivity analyses are discussed in the supporting Text S1.

### Predictions of simpler models

**Figure 7:**
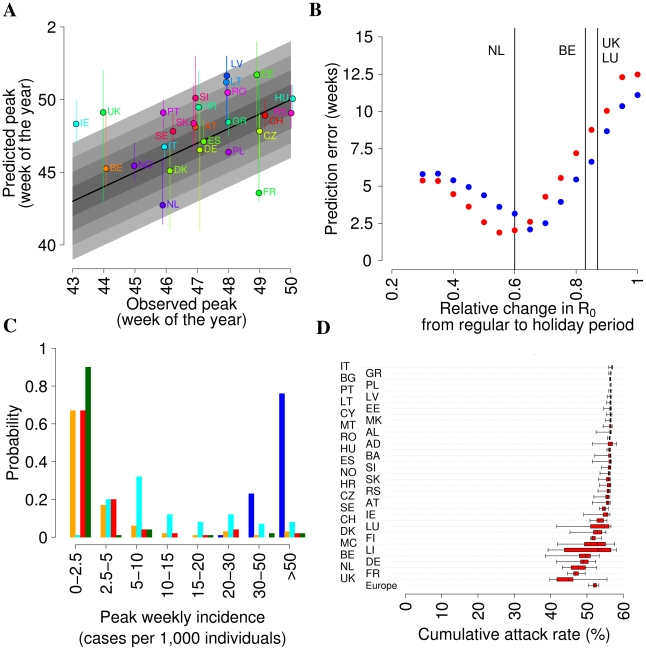
Predictions of simpler models (*R_0_* = 1.48 *T_g_* = 3.1 days). (A) Observed peak week plotted versus predicted peak week (predictions refer to the best model with coupling between European countries: *R*_0_ = 0.8 during holidays; vertical bars represent 95% confidence intervals of the predictions) for European countries covered by the WHO/Europe weekly influenza surveillance system; only the Autumn wave is considered for UK. (B) Prediction error (average of the absolute value of predicted minus observed peak week in European countries covered by the WHO/Europe weekly influenza surveillance system) as a function of the relative change of *R_0_* during the summer for models with (red points) and without (blue points) coupling between European countries (i.e. long-distance travel). Vertical lines corresponds to the relative change of *R*_0_ during holidays in four European countries as resulting from the analysis of the POLYMOD data [39]. (C) Probability of observing a summer wave with peak incidence in a given range (predictions refer to the best model with coupling between European countries: *R*_0_ = 0.8 during holidays), in UK (blue), Germany (cyan), Netherlands (orange), Ireland (green) and Spain (red). (D) Cumulative attack rate (2.5%, 25%, 50%, 75% and 97.5% percentiles of the distribution are shown) in the different European countries (predictions refer to the best model with coupling between European countries: *R_0_* = 0.8 during holidays). A total of 100 simulations for each parameter set were undertaken to produce the results shown.

Given the computational requirements of large scale simulations, a relevant question is the extent to which such a model is required to reproduce the epidemiological patterns seen in pandemic spread across Europe. We therefore also considered model variant which substitutes the individual based spatial simulation of transmission within European countries with a simple stochastic homogeneous mixing model, while retaining other aspects of the original model; *i.e.* importation of cases from United States and Mexico, inter country long-distance travel, realistic school calendars, length of latent and infectious periods (1.5 days and 1.6 days respectively), and *R*_0_ (1.48 in all countries). Details are reported in the supporting Text S1. The relative change of *R*_0_ during holidays (to simulate school closure) was optimized to fit the autumn peak week in the different European countries. As shown in Fig. 7A, predictions of the autumn peak week in the different European countries are consistent with observed data. However, some limitations of this approach are revealed: firstly, predictions are very sensitive to the change in *R*_0_ during holidays, and the optimal value of this parameter was difficult to predict before the end of the epidemic, thus making it very difficult to use such a model for real-time prediction. In addition, as shown in Fig. 7B, the relative change of *R*_0_ during holidays which gives an optimal fit to the timing of the epidemics in Europe is different from values suggested in the literature [39]. Moreover, Fig. 7C shows that the model fails to predict the dynamics of the epidemic in UK (where a single very large summer wave is predicted, with little or no autumn wave) and other European countries: for instance, a summer wave is consistently predicted in Germany, probably because of the lack of demographic variation in the model; in fact, as shown above, it is possible that the epidemic spread was delayed in Germany during the summer because of its older population. Finally, Fig. 7D shows that the model predicts cumulative attack rates of about 57% (except for countries, like UK, France and Germany, where the effect of school closure on final size is larger) which is quite unrealistic but is a known consequence of models with homogeneous mixing. More realistic results might be obtainable by including heterogeneous mixing with age, but the resulting model would then have comparable (or larger) numbers of transmission parameters as our individual simulation.

## Discussion

Our analysis has shown that a spatially explicit transmission model gives a broadly realistic depiction of the spatiotemporal spread of 2009 pandemic flu through Europe and insight into the main factors explaining the geographical differences in transmission dynamics. Furthermore, we have shown that the model is sufficiently robust that a parameterisation on the basis of incomplete data collected up to the end of June suffices for the model to predict patterns of spread broadly in agreement with observations. However, the use of some pandemic-specific data in model parameterisation was essential – most notably in calibrating epidemic growth rates and representing variation of susceptibility to infection with age.

In particular, we have demonstrated that the substantial summer wave of transmission seen only in the UK only was not a chance effect, or a phenomenon needing special explanation, but emerges as an almost inevitable consequence of travel patterns from US and Mexico to different European countries [23], of the timing of virus emergence in Mexico, and of school calendars across Europe. An interesting comparison is with Germany which did not differ greatly from UK in terms of number of imported cases in the initial phase of the epidemic and the timing of school closure (though holiday timing is more variable within Germany). However, while the baseline simulations of the model predict a large summer wave in UK, only a few simulations show a relevant summer wave in Germany while in the large majority of them very little community transmission is predicted during the summer, consistently with observed data [2]. This results partly from the somewhat lower German import rate and earlier school holidays, but is also due the fact that Germany has one of the oldest populations in Europe and, as an effect of differential susceptibility to infection, this reduces transmission rates (Fig. 3A).

Another prediction of the model is that the final infection attack rate varied significantly from country to country as a consequence of differences in sociodemographic structure explicitly accounted for in the model; in particular, we found that the attack rate is positively correlated with the average household size and negatively with mean age of a population (Table 2). These predictions can be tested, if data from serological surveys in several countries become available. In Norway, for instance, recently published data show that the frequency of protective antibodies was particularly high in persons under 20 years of age (61.2%, 95% CI 53,6%–68,8%) while in people aged 20 years and older the figures were substantially lower (37.8%, 95% CI 32.8%–42.8%) [40]. However, in the case of Norway it is difficult to discriminate effects of infection and vaccination (vaccination in Norway started in mid-October 2009 and population vaccine uptake probably exceeded 40%), and this has already been argued as possibly explaining the relatively high seroconversion rates seen in adults [40]. In a few countries a significant difference exists between the observed and predicted week of peak incidence, most notably for France, where the peak is predicted on average at week 43.6, while it actually occurred at week 49. As discussed above, the timing of school holidays (weeks 44 and 45) in France was such that small differences in parameter values could cause large difference in peak week. There were also regional differences in peak week (peak in the Paris region, the best connected to the rest of the world, occurred at week 44). Several other factors not included in the model, may have influenced the dynamics in some countries: reactive school closures (as occurred in a scattered manner in France, while in the whole country for a couple of weeks in Bulgaria), seasonality in travel, other viruses (e.g. Rhinoviruses and RSV) potentially interfering with influenza transmission or detection [41], [42]; climate [8], [43]–[45] (though no obvious pattern is detectable, see supporting Text S1); and the effect of coupling to neighbouring countries (for instance Ukraine and Russia) where a sizeable epidemic was observed around the same period.

The model used has several other limitations; commuter travel patterns within Europe were modelled from data available only from few countries, while in reality heterogeneity in travel behaviour is probably extensive. In addition, changes in contact behaviour during school holidays are represented very simply [12]. It is to be expected that incorporating more information in the model would improve its accuracy. For instance, large differences in incidence were seen in the summer wave from region to region in England [33]. Our simulation also predicted extensive spatial heterogeneity within countries at that time: realisations corresponding to the top of the prediction band of Fig. 2A give widespread infection across the UK by July 20, while in realisations contributing to the bottom of the prediction band of Fig. 2A, foci of infection were present in some but not all large cities within the UK by mid-July. Making more rigorous comparisons of within-country variation was beyond the scope of the current study, but we would expect it to be important to use age-dependent country-specific mobility data (which was not available for this work), and to account for within-country demographic heterogeneity (both factors that likely affected the within-country timing of spread) in order to accurately match patterns of local spread.

However, as shown by the sensitivity analyses presented above and in the SI, the main conclusions of our work seem to be robust. So long as the model includes the timing of imported cases into different countries, realistic school calendars and basic information on mobility and demography, when R_0_ and T_g_ are in a (relatively small) region consistent with UK epidemic growth rate estimates available in June, the model predicts with high likelihood that a summer wave would occur in UK only, and a timing of autumn waves in different countries with good correspondence to the observed pattern.

The model used may appear relatively complex, and leading one to consider whether simpler models could yield similar qualitative patterns. As shown by the comparisons in Fig. 7, a simple compartmental SEIR model with spatial structure, if appropriately parameterised, can reproduce the timing of autumn epidemic peak almost as well as the model used in this study. However, this requires careful fine-tuning of the assumed reduction in transmission caused by school holidays, which would be difficult to undertake early in a pandemic, given that the optimal value we estimated for this parameter differs from published values [39]. Furthermore, simpler models fail to reproduce the observed distribution of sizes of the summer wave in European countries, and predict high attack rates. It is possible that, adding age-dependent contact rates and differential age structure between countries, one could improve the overall fit, but this would introduce multiple additional parameters.

The model under study has actually only 6 free epidemiological parameters, namely the transmission coefficients in households, schools, workplaces, the wider community and during long-distance travel, and the susceptibility of adults relative to children. Note that we assume these parameters are constant across Europe; differences in country-specific value of *R*_0_ arise as a consequence of the different sociodemographic structure of countries' populations. We actually only tuned 1 free parameter, a scale parameter allowing simulating epidemics with different values of R_0_^UK^, with pre-pandemic estimates being used to parameterise transmission in households, and estimates of variation of susceptibility with age being derived from data available early in the 2009 pandemic [23]. The underlying model structure just uses data on the natural history of the virus (informing incubation period and generation time estimates) and the social structure and demography of the population without any post-hoc fitting to the 2009 data.

Thus while the state space of the model is high dimensional, the parameter space is (like previous models of this type [16], [17]) low dimensional and certainly no more over-parameterised than a traditional age-structured compartmental patch model with coupling between the different countries. Of course, the structural assumptions of the model – namely how space and social structure are represented - are open to challenge (as for all models), but the relative success the model achieves in matching the heterogeneous spatiotemporal dynamics of the 2009 pandemic without the need to fine tune large numbers of parameters offers a degree of comfort that the assumptions made are reasonable.

By comparing model predictions with those of the pre-pandemic model in ref. [10] it is readily apparent that the latter, by failing to take account of differences in susceptibility between adults and children, would have overestimated the final attack rate, especially in adults, with the resulting predictions being similar to those shown in Fig. 6. Moreover, by not considering the effects of school holidays on transmission, most pre-pandemic models would have failed to correctly predict two waves in UK and a single autumn/winter wave in all other European countries. Overall, the prediction of pandemic timing would have been even worse than those shown in Fig. 5. This highlights the requirement for models to be carefully re-parameterised using data collected in real-time in an emerging epidemic.

A relevant question is whether the availability of these modelling techniques might be helpful in designing and implementing control policies in the face of a new flu pandemic. This work shows that a model of this type, that takes into account transmission in different social contexts, mobility patterns and demographic information, may provide useful estimates of the timing of infection spread, using limited epidemiological information such as might be available early in a pandemic. A future pandemic can be expected to have different transmission characteristics from the 2009 virus, so that the model described here would need to be reparameterised before application. However, it has been shown here that a good estimate of exponential growth rate in the countries that first experience the infection, together with an assessment of the relative susceptibility of different age classes, may suffice to provide a good prediction of epidemic timing, independently of many other small details.

Our results show that another crucial requirement is obtaining reliable estimates of the number of imported cases over time in the different countries during the initial phase of the epidemic. It is possible, however, that such data will be not readily available for the next pandemic (e.g. the epidemic could spread much faster than the 2009 H1N1 pandemic) and thus a simplified procedure will be needed for estimating imported cases over time in the different countries. Our results suggest (see SI) that using airline data on passenger volumes would still give reasonable (though slightly less accurate) results.

We have not considered the issue of clinical severity (*e.g.* case fatality ratios) in this paper. As of June 2009 (when vaccine purchasing decisions were being made by many countries), data on severity was still very limited, with the upper bound on case fatality estimates still being in the region of those estimated for the 1957 and 1968 pandemics [23], [46]. Only once data became available from the Southern Hemisphere countries after their initial epidemics were over (late August 2009), were more accurate estimates of severity able to be made [47]. Definitely, a key lesson learnt from the 2009 pandemic is the necessity to improve tools for obtaining early estimates of severity. The present paper does not address this issue, but, if information on severity were obtained, these could be incorporated into this (or similar) model to provide predictions quantified in terms of expected hospitalization rates, intensive care unit admissions and mortality.

Estimates on the expected course and timing of country-specific epidemics of the type resulting from the model presented here, together to information about the likely time of availability of a vaccine, would be helpful in optimising vaccination campaign design. For instance, where vaccine was predicted to become available well before the epidemic peaked, it might be targeted at groups most responsible for transmission (e.g., school-age children), while when vaccine was only predicted to be available towards the peak of transmission (as was the case in, for instance the UK in 2009), targeting groups at highest risk of severe clinical outcomes might be preferred. In a more severe pandemic, one could also examine whether deliberate school closure policies [48], or aggressive antiviral prophylaxis, aiming at delaying the epidemic peak, might be effective at delaying peak timing sufficiently to allow vaccination to be undertaken.

More generally, we believe that, if these modelling results, validated by some previous experience, had been available by June-July 2009, they could have reduced uncertainty and improved situational awareness for policy-makers across Europe, and given rather clearer expectations as to the likely impact (albeit not in terms of mortality) and timing of the pandemic. As such, we believe that the work presented here supports the use of this type of modelling for assessing in real time the likely effects of future flu pandemics and for evaluating mitigation measures.

## Supporting Information

Text S1 Supplementary methods and results.
(PDF)

## References

[1] Khan K, Arino J, Hu W, Raposo P, Sears J (2009). Spread of a Novel Influenza A (H1N1) Virus via Global Airline Transportation.. N Engl J Med.

[2] Poggensee G, Gilsdorf A, Buda S, Eckmanns T, Claus H (2010). The first wave of pandemic influenza (H1N1) 2009 in Germany: from initiation to acceleration.. BMC Infect Dis.

[3] Hahné S, Donker T, Meijer A, Timen A, van Steenbergen J et al (2009). Epidemiology and control of influenza A(H1N1)v in the Netherlands: the first 115 cases.. Euro Surveill.

[4] Gilsdorf A, Poggensee G, Working Group Pandemic Influenza A(H1N1)v (2009). Influenza A(H1N1)v in Germany: the first 10,000 cases.. Euro Surveill.

[5] Belgian working group on influenza A(H1N1)v (2009). Influenza A(H1N1)v virus infections in Belgium.. Euro Surveill.

[6] World Health Organization (2010). http://www.euro.who.int/__data/assets/pdf_file/0003/91839/E93581.pdf.

[7] Paget J, Marquet R, Meijer A, van der Velden K (2007). Influenza activity in Europe during eight seasons (1999–2007): an evaluation of the indicators used to measure activity and an assessment of the timing, length and course of peak activity (spread) across Europe.. BMC Infect Dis.

[8] Shaman J, Pitzer VE, Viboud C, Grenfell BT, Lipsitch M (2010). Absolute humidity and the seasonal onset of influenza in the continental United States.. PLoS Biol.

[9] Colizza V, Barrat A, Barthelemy M, Valleron A-J, Vespignani A (2007). Modeling the Worldwide Spread of Pandemic Influenza: Baseline Case and Containment Interventions.. PLoS Med.

[10] Merler S, Ajelli M (2010). The role of population heterogeneity and human mobility in the spread of pandemic influenza.. Proc Biol Sci.

[11] Dushoff J, Levin S (1995). The effects of population heterogeneity on disease invasion.. Math Biosci.

[12] Cauchemez S, Valleron A, Boëlle P, Flahault A, Ferguson NM (2008). Estimating the impact of school closure on influenza transmission from Sentinel data.. Nature.

[13] Chao DL, Halloran ME, Longini IM (2010). School opening dates predict pandemic influenza A(H1N1) outbreaks in the United States.. J Infect Dis.

[14] Longini IM, Halloran ME, Nizam A, Yang Y (2004). Containing pandemic influenza with antiviral agents.. Am J Epidemiol.

[15] Longini IM, Nizam A, Xu S, Ungchusak K, Hanshaoworakul W (2005). Containing pandemic influenza at the source.. Science.

[16] Ferguson NM, Cummings DAT, Cauchemez S, Fraser C, Riley S (2005). Strategies for containing an emerging influenza pandemic in Southeast Asia.. Nature.

[17] Ferguson NM, Cummings DAT, Fraser C, Cajka JC, Cooley PC (2006). Strategies for mitigating an influenza pandemic.. Nature.

[18] Germann TC, Kadau K, Longini IM, Macken CA (2006). Mitigation strategies for pandemic influenza in the United States.. Proc Natl Acad Sci USA.

[19] Ciofi degli Atti ML, Merler S, Rizzo C, Ajelli M, Massari M (2008). Mitigation measures for pandemic influenza in Italy: an individual based model considering different scenarios.. PLoS ONE.

[20] Halloran ME, Ferguson NM, Eubank S, Longini IM, Cummings DAT (2008). Modeling targeted layered containment of an influenza pandemic in the United States.. Proc Natl Acad Sci USA.

[21] Gojovic MZ, Sander B, Fisman D, Krahn MD, Bauch CT (2009). Modelling mitigation strategies for pandemic (H1N1) 2009.. CMAJ.

[22] Ajelli M, Merler S, Pugliese A, Rizzo C (2011). Model predictions and evaluation of possible control strategies for the 2009 A/H1N1v influenza pandemic in Italy.. Epidemiol.

[23] Fraser C, Donnelly CA, Cauchemez S, Hanage WP, Kerkhove MDV (2009). Pandemic potential of a strain of influenza A(H1N1): early findings.. Science.

[24] Ghani AC, Baguelin M, Griffin J, Flasche S, Pebody R (2009). The Early Transmission Dynamics of H1N1pdm Influenza in the United Kingdom.. PLoS Curr.

[25] Eurostat (2011). http://epp.eurostat.ec.europa.eu/portal/page/portal/statistics/search_database.

[26] European Centre for Disease Prevention and Control (2009). http://reliefweb.int/sites/reliefweb.int/files/resources/407CA95824E598C3852575CA0062F80A-Full_Report.pdf.

[27] Cauchemez S, Donnelly CA, Reed C, Ghani AC, Fraser C (2009). Household transmission of 2009 pandemic influenza A (H1N1) virus in the United States.. N Engl J Med.

[28] Hippisley-Cox J, Smith S, Smith G, Porter A, Heaps M (2006). QFLU: new influenza monitoring in UK primary care to support pandemic influenza planning.. EuroSurveill.

[29] Health Protection Agency (2009). http://www.hpa.org.uk/webc/HPAwebFile/HPAweb_C/1246433639498.

[30] Wallinga J, Lipsitch M (2007). How generation intervals shape the relationship between growth rates and reproductive numbers.. Proc Biol Sci.

[31] Cowling BJ, Fang VJ, Riley S, Malik Peiris JS, Leung GM (2009). Estimation of the Serial Interval of Influenza.. Epidemiology.

[32] World Health Organization. Situation updates - Pandemic (H1N1) (2009). http://www.who.int/csr/disease/swineflu/updates/en/index.html.

[33] Miller E, Hoschler K, Hardelid P, Stanford E, Andrews N (2010). Incidence of 2009 pandemic influenza A H1N1 infection in England: a cross-sectional serological study.. Lancet.

[34] Hardelid P, Andrews NJ, Hoschler K, Stanford E, Baguelin M (2010). Assessment of baseline age-specific antibody prevalence and incidence of infection to novel influenza AH1N1 2009.. Health Technol Assess.

[35] Istituto Superiore di Sanità (2010). http://www.epicentro.iss.it/focus/h1n1/pdf/flunews/FluNews_17.pdf.

[36] Italian Ministry of Health (2010). http://www.salute.gov.it/influenza/documenti/virologica/AggVir_05-05-10.pdf.

[37] Réseau Sentinelles FranceINSERMUPMC (2010). http://www.sentiweb.fr.

[38] Bootsma MCJ, Ferguson NM (2007). The effect of public health measures on the 1918 influenza pandemic in U.S. cities.. Proc Natl Acad Sci USA.

[39] Hens N, Ayele GM, Goeyvaerts N, Aerts M, Mossong J et al (2009). Estimating the impact of school closure on social mixing behaviour and the transmission of close contact infections in eight holidays regular European countries,. BMC Infect Dis.

[40] Waalen K, Kilander A, Dudman SG, Krogh GH, Aune et al (2010). High prevalence of antibodies to the 2009 pandemic influenza A(H1N1) virus in the Norwegian population following a major epidemic and a large vaccination campaign in autumn 2009.. Euro Surveill.

[41] Casalegno JS, Ottmann M, Duchamp MB, Escuret V, Billaud G (2009). Rhinoviruses delayed the circulation of the pandemic A (H1N1) 2009 virus in France.. Clin Microbiol Infect.

[42] Linde AM, Rotzen-Ostlund M, Zweyberg-Wirgart B, Rubinova S, Brytting M (2009). Does viral interference affect spread of influenza?. Euro Surveill.

[43] Shaman J, Kohn M (2009). Absolute humidity modulates influenza survival, transmission, and seasonality.. Proc Natl Acad Sci USA.

[44] Balcan D, Hu H, Goncalves B, Bajardi P, Poletto C (2009). Seasonal transmission potential and activity peaks of the new influenza A(H1N1): a Monte Carlo likelihood analysis based on human mobility.. BMC Med.

[45] Truscott J, Fraser C, Hinsley W, Cauchemez S, Donnelly C (2009). Quantifying the transmissibility of human influenza and its seasonal variation in temperate regions.. PLoS Curr.

[46] Garske T, Legrand J, Donnelly CA, Ward H, Cauchemez S (2009). Assessing the severity of the novel influenza A/H1N1 pandemic.. British Med J.

[47] Baker MG, Wilson N, Huang QS, Paine S, Lopez L (2009). Pandemic influenza A(H1N1)v in New Zealand: The experience from April to August 2009.. Euro Surveill.

[48] Wu JT, Cowling BJ, Lau EHY, Ip DKM, Ho LM (2010). School Closure and Mitigation of Pandemic (H1N1) 2009, Hong Kong.. Emerg Infect Dis.

