# Supplementary material for: Determinants of the Spatiotemporal Dynamics of the 2009 H1N1 Pandemic in Europe: Implications for Real-Time Modelling
Source: PLoS Comput Biol. 2011 Sep 29;7(9):e1002205. doi: 10.1371/journal.pcbi.1002205 (PMC3182874; doi:10.1371/journal.pcbi.1002205)
Supplement: Text S1 — Supplementary methods and results. (PDF) [file pcbi.1002205.s001.pdf]

Determinants of the spatiotemporal dynamics of the 2009 H1N1 pandemic in Europe: implications for real-time modelling

Stefano Merler[1], Marco Ajelli[1], Andrea Pugliese[2] and Neil M. Ferguson[3]

[1] Bruno Kessler Foundation, via Sommarive 18, I-38123 Trento, Italy
[2] Mathematics Department, University of Trento, via Sommarive 14, I-38123 Trento, Italy
[3] MRC Centre for Outbreak Analysis and Modelling, Department of Infectious Disease Epidemiology, Imperial College London, Faculty of Medicine, Norfolk Place, London W2 1PG, UK

Supporting Text S1

Contents

1 Methods **3**
1.1 Travel–related cases . . . 3
1.2 Imported cases . . . 4
1.3 Transmission model . . . 9
1.4 Natural history of influenza and model parametrization . . . 11
1.5 Computation of R_0 . . . 18
1.6 Realizations and resources . . . 19

2 Climatic factors **19**

3 Predictions of homogeneous mixing models **20**

4 Sensitivity analysis **22**
4.1 Age-dependent susceptibility to infection . . . 22
4.1.1 Susceptibility of children relative to adults: 4/1 . . . 22
4.1.2 Susceptibility of children relative to adults: 1/1 . . . 22
4.2 Sociodemographic structure . . . 23
4.3 Patters of human mobility . . . 25
4.3.1 Inter–European travel . . . 25
4.3.2 Importation of cases from US and Mexico . . . 26
4.4 School closure . . . 27
4.4.1 School closure in Europe . . . 27
4.4.2 School closure in United States . . . 28
4.5 Workplaces . . . 29
4.6 Case detection . . . 30

Bibliography **31**

1 Methods

The model is an individual-based stochastic simulation model structurally similar to a model previously developed for Europe (Merler and Ajelli, 2010). The simulation is a spatially-explicit discrete-time SEIR model with force of infection decreasing with the geographical distance which explicitly models transmission in households, schools and workplaces. Infection spread between countries is modelled through cross-border diffusion and long-distance travel. As for the details we refer the reader to (Merler and Ajelli, 2010).

Briefly, the Gridded Population of the World version 3 (GPW v3) (Balk and Yetman, 2004) produced by the Center for International Earth Science Information Network (CIESIN) of the Earth Institute at Columbia University was used as the source of population density for the study area. The study area covers 37 European countries and includes all the member states of the European Union. The total population is about 515 million individuals, divided in 63,794 cells, whose average surface is about 77 square kilometers. Sociodemographic data (age structure, frequencies of household type and size, employment rate, etc.) provided by the Statistical Office of the European Communities (Eurostat) and integrated with data provided by the National Statistical Offices for countries not covered by Eurostat were used to allocate individuals in households and to assign an employment to individuals. An heuristic model which matches marginal distributions of household size and population age structure, and maintains realistic generational age gaps within households (by avoiding randomly assigned ages to the households members), respecting as best as possible the actual mix of age groups was used to generate a realistic model of households. Data from the PIRLS 2001 and PISA 2000 and 2003 international surveys (European Commission, 2005) were used to determine country-specific distributions of school size and to locate students in schools. Data on the distribution of the workplaces size in Italy and UK were used for assigning the size to the workplaces in all the countries of the study area. Long-distance travel were modelled making use of European air and railway transportation data as provided by Eurostat.

In the following of this Section, we discuss in detail only features that are specific for this study.

1.1 Travel–related cases

The analysis of the first 711 laboratory-confirmed cases of H1N1pdm influenza in Europe available at June 3 with individual case reports revealed that 452 of them were presumably been infected abroad (European Centre for Disease Prevention, 2009). The countries with the larger cumulative number of confirmed cases were UK (339), Spain (180), France (42), Germany (37) and Italy (30). The percentage of cases related to travels in these countries was 53% in UK , 53% in Spain, 98% in France, 81% in Germany and 93% in Italy. Overall, the percentage of imported cases in Europe was about 64%. Mexico and US, the two countries affected earlier by the H1N1pdm influenza strain, account for 69% and 21% of infections from abroad respectively (European Centre for Disease Prevention and Control, 2009). These data allow for a rough computation of the probability p_c of importing cases in the different countries c, namely $p_c = zI_cN_c$ where N_c is the number of confirmed cases in country c up to June 3, I_c is the estimated fraction of travel–related cases in country c and z is a normalization factor.

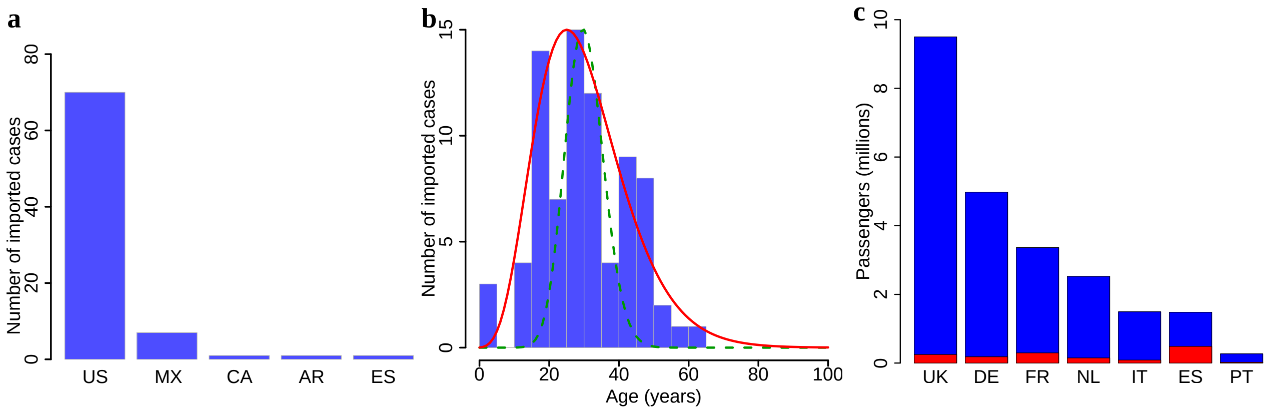

Figure S1: **a** Suspected country of provenience (MX: Mexico; CA: Canada; AR: Argentina; ES: Spain) of imported cases as resulting from the analysis of the first 152 laboratory-confirmed cases of H1N1pdm influenza in Italy. **b** Age of imported cases in Italy. In the model, to assign an age to imported cases we used a negative binomial distribution of mean 30.2 and probability 0.1772 (red line, compared with a Poisson distribution, dashed green line). **c**. Yearly number of passengers entering different European countries from US (blue) and Mexico (red).

Later works, providing estimates of the fraction of imported and based on data collected up to July 7, confirmed these early findings. In Italy, the analysis of the first 152 laboratory-confirmed cases of H1N1pdm influenza in Italy revealed that 86% of them presumably had been infected abroad, primarily either in US or Mexico, the two countries affected earlier by the H1N1pdm influenza strain (these two countries account for 84% and 8% of infections from abroad respectively, see Fig. S1a) and infection was found predominantly in relatively young individuals (the age of imported cases ranges from 0 to 61 years with an average of 30 years, see Fig. S1b) (Rizzo et al., 2009). In the Netherlands, only 3 cases were imported by the end of May and, by 24 June, 115 confirmed cases were reported, of whom 64 (56%) were most likely imported and 51 (44%) were indigenously acquired (Hahn et al., 2009). In Belgium, among the first 43 laboratory-confirmed cases, infection was acquired abroad by 35 cases, of which 18 had a travel history to the US (Belgian working group on influenza A(H1N1)v, 2009). In Germany, while in the beginning most cases were imported, the proportion of indigenous cases has increased since 2 June 2009. Of the 190 confirmed cases, 93 cases (47%) were imported (Novel influenza A(H1N1) investigation team, 2009).

By analyzing air transportation data as provided by Eurostat, we found that in 2007 nearly 27 million passengers have entered EU27 from US (about 25.5 millions) and Mexico (about 1.5 millions). Overall, the great majority of such trips were to the Western part of Europe (see Fig. S1c). Specifically, about one third of such trips were to UK and, remarkably, about one third of trips from Mexico were to Spain.

1.2 Imported cases

We used a discrete-time stochastic SEIR epidemic model for simulating cases imported over time from Mexico and US. The number of imported cases at each time step Δt of the simu-

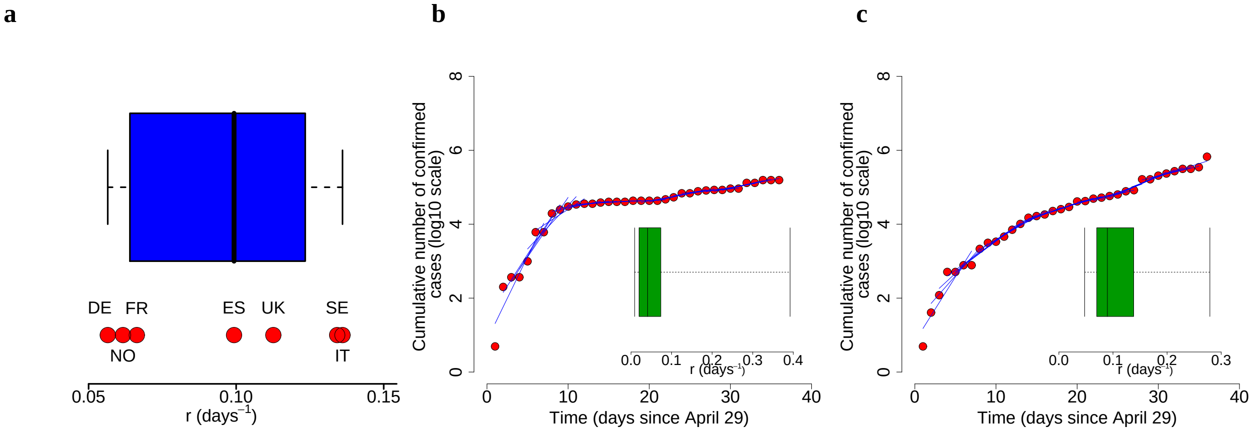

Figure S2: **a** Distribution (minimum, 25%, 50%, 75% percentiles and maximum) of the growth rate r (red points indicate country–specific values) of the number of confirmed cases in some European countries. **b** For each country, r is computed by averaging growth rates computed week by week up to June 3. Here we show the procedure for Spain. In the inset 2.5%, 25%, 50%, 75% and 97.5% percentiles of the distribution of r. **c** As **b** but for UK.

lation in each European country c is assumed to be a sample from a Poisson distribution of parameter

$$p_c T \Delta t [E(t) + I(t)]/\tilde{N} \tag{1}$$

where $E(t)$ and $I(t)$ denote the number of predicted exposed and infected individuals at time t in US and Mexico; $\tilde{N}$ the population of US and Mexico, T is the daily number of travellers entering Europe from US and Mexico, p_c is the probability of importing cases in country c from US and Mexico as resulting from the analysis reported in Sec. 1.1. Finally, imported cases are randomly assigned to a cell of the country (by replacing susceptible individuals) with probability proportional to the population.

The homogeneous SEIR model is parametrized through the length of the latent period $1/\omega$, the length of the infection period $1/\gamma$ and the transmission rate β.

Latent and infectiousness periods have the same duration as in the individual based model (see Sec. 1.4 paragraph Generation time). For SEIR models, the reproduction number R_0, defined as the average number of cases that results in a fully susceptible population from a single infectious individual over the course of his infectious period (Anderson and May, 1992), is

$$R_0^{ode} = \frac{\beta}{\gamma} \, . \tag{2}$$

The exponential growth rate r is the larger solution of the characteristic equation:

$$r^2 + (\omega + \gamma) r + (\gamma - \beta)\omega = 0 \, . \tag{3}$$

By solving Eq. (3) for the transmission rate β and by substituting the resulting value of β in Eq. (2) one obtains the following expression for R_0, depending only on r and the lengths of

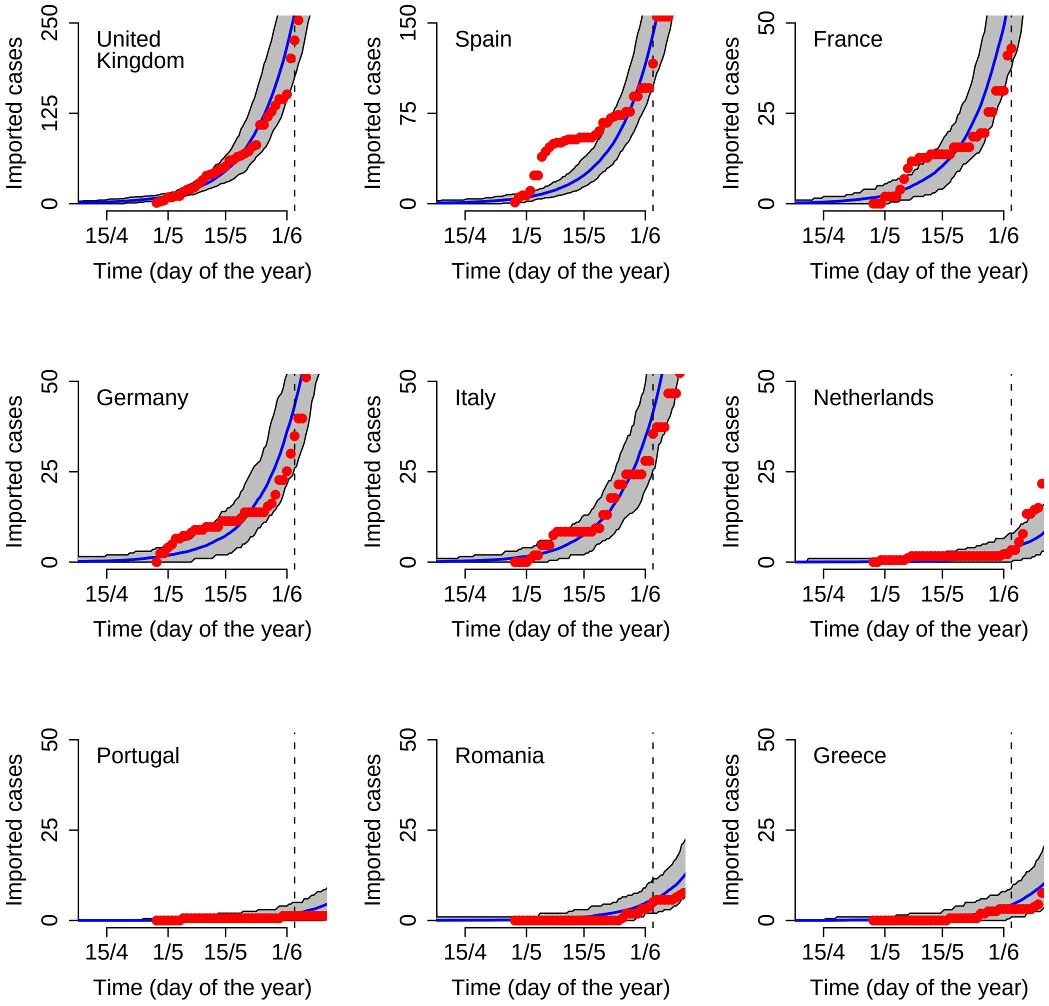

Figure S3: Average cumulative number of imported cases over time in different model realizations ($R_0^{UK} = 1.48$, $T_g = 3.1$ days) in some European countries (blue lines) and 95% confidence intervals (grey areas) plotted against the rescaled cumulative number of confirmed cases (red points). Early estimates of the fraction of imported cases in the first phase of the epidemic were available for a few countries, namely 53% in UK and Spain, 98% in France, 81% in Germany and 93% in Italy. For other countries we assume that the percentage of imported cases is 64%, i.e. the European ratio of travel related cases to the number of confirmed cases. Red points represent the rescaled cumulative number of confirmed cases and thus represent a rough estimate of the cumulative number of imported (confirmed) cases over time.

latent and infectious periods:

$$R_0 = \left(1 + \frac{r}{\gamma}\right) \cdot \left(1 + \frac{r}{\omega}\right) \tag{4}$$

We estimated the growth rate r of the number of confirmed cases in some European

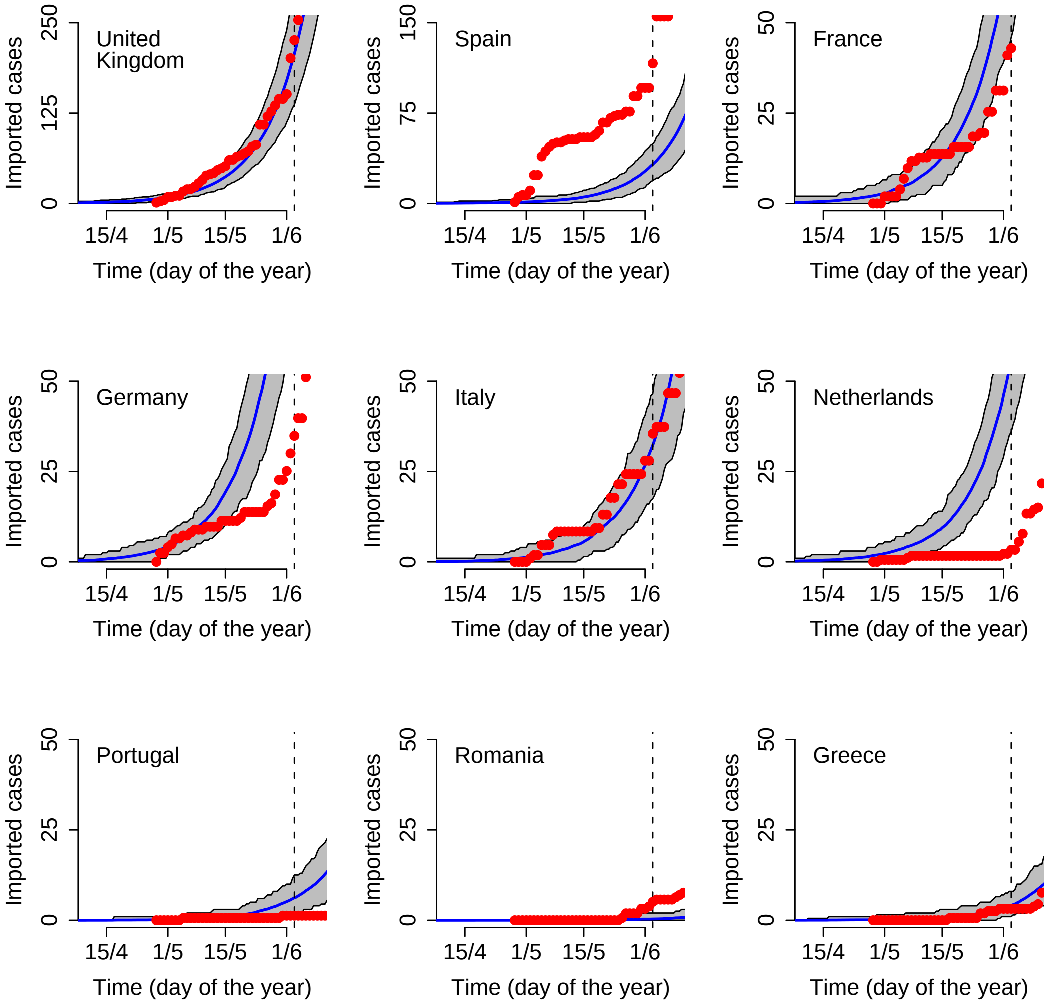

Figure S4: Average cumulative number of imported cases over time in different model realizations ($R_0^{UK} = 1.48$, $T_g = 3.1$ days) in some European countries by assuming that imported cases are proportional to the daily number of travellers from US and Mexico (blue lines) and 95% confidence intervals (grey areas) plotted against the rescaled cumulative number of confirmed cases (red points, see Fig. S3).

countries (where there is a measurable growth, e.g. UK, France, Germany) in the initial phase of the epidemic (up to June 3), obtaining a median value $\bar{r} = 0.099$ days^{-1} (standard deviation SD=0.03, see Fig. S2). From (4) we obtain $R_0 = 1.33$ assuming latent period 1.5 day and infectious period 1.6 days (the baseline assumption; see Sec. 1.4 paragraph Generation time), a value compliant with estimates for Mexico (Fraser et al., 2009). When we change the generation time, we obtain a different estimate for R_0 to match the observed growth rate $\bar{r}$. For instance, values of R_0 range from 1.28 for $T_g = 2.7$ days to 1.38 for $T_g = 3.5$ days.

We consider that importation of cases was reduced during summer holidays in US (from

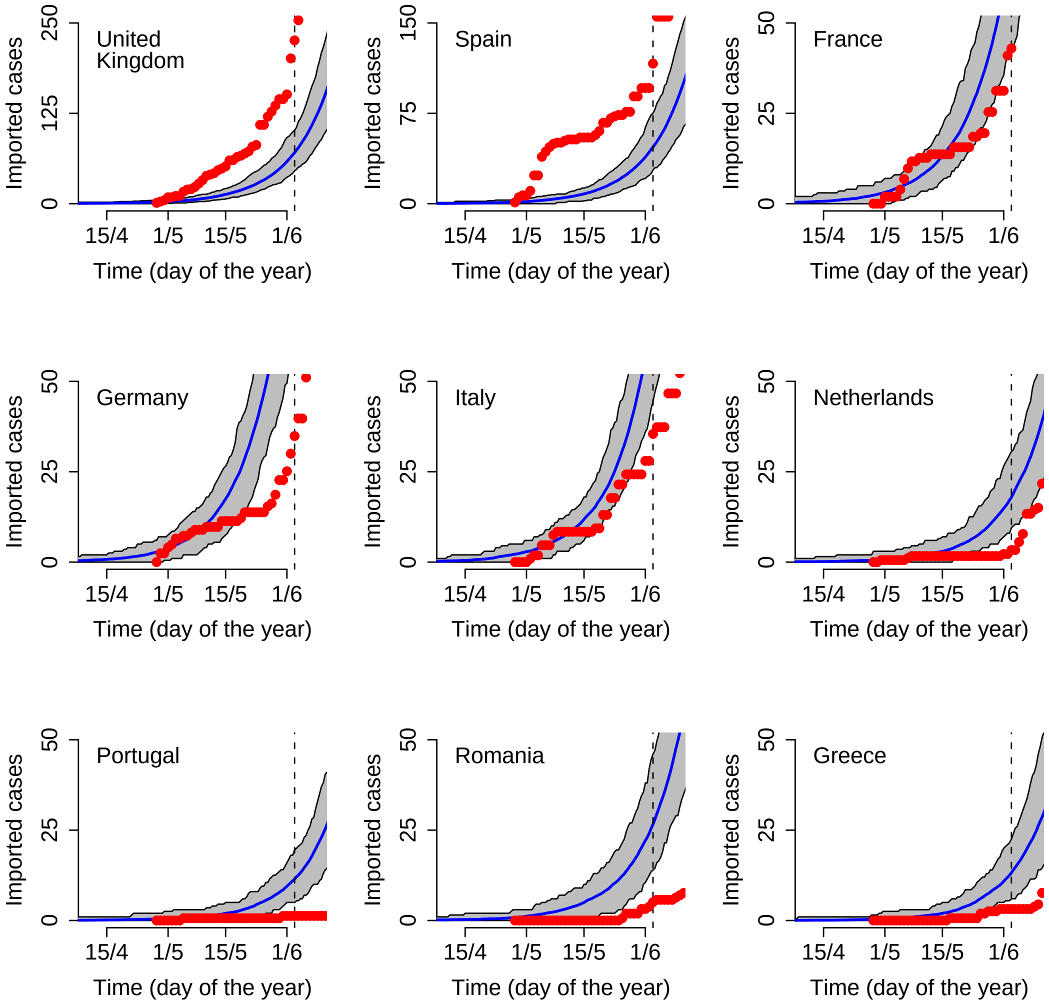

Figure S5: Average cumulative number of imported cases over time in different model realizations ($R_0^{UK} = 1.48$, $T_g = 3.1$ days) in some European countries by assuming that imported cases are proportional to the population (blue lines) and 95% confidence intervals (grey areas) plotted against the rescaled cumulative number of confirmed cases (red points, see Fig. S3).

June 15 to August 15; a much more complex model would have been required to account for heterogeneity in the timing of summer holidays in US); in this period R_0 drops to 0.8. Some variants of this assumption are also evaluated (see Sec. 4.4.2).

Eq. (1) thus allows simulating importation of cases from US and Mexico into the different European countries at the rate observed in the data. We stress the fact that we set R_0 in the SEIR model to reproduce this pattern rather than to estimate R_0 for the epidemic in Mexico and US (which is beyond the aim of this paper), though we obtained estimates of R_0 compliant with early estimates for Mexico. In fact, we do not aim to estimate the spread of the epidemic in US and Mexico; rather, we only need a consistent procedure to estimate

importation of cases over time in the different European countries.

Simulated imported cases are shown in Fig S3; they are quite consistent with observed data. In a variant of the model, we assumed that $p_c T$ in (1) would represent the number of daily travellers from US and Mexico to the European country c, as resulting from the analysis of airline data (see Fig. S4). The model would have failed to correctly predict the number of imported cases in Spain, as the model tends to reproduce better the importation of cases in countries with strong connections with US (because of the much larger connection US-Europe with respect to Mexico-Europe). Moreover, the model would have overestimated the number of imported cases in Germany and the Netherlands, probably because flights to the airport hubs of Amsterdam, Frankfurt and Munich do not necessarily represent the final destination of passengers. In another variant of the model, we assumed that the constant p_c in (1) is proportional to the population size (see Fig. S5). This model would have completely failed to correctly predict the number of imported cases in the different countries. These variants of the model are discussed in detail in Sec. 4.3.2.

Finally, we remark that Eq. (4) can be used for estimating R_0 also for observed data (see Sec. 1.4 paragraph R_0) and for epidemics simulated through the individual based model (see Sec. 1.5).

1.3 Transmission model

The influenza transmission within Europe was simulated by a stochastic spatially structured individual–based model. We considered an epidemic occurring within the European population, accounting for ≈ 515 million individuals, kept constant during a simulation (i.e., without considering neither born/dead nor immigration/emigration processes). Individuals are explicitly represented in the model and are characterized by citizenship, household membership, school and workplace membership (if any). Households, schools and workplaces are located in an explicit geographic location.

Once the population is initialized, at any time t of the simulation (time step $\Delta t = 0.5$days), any susceptible individual i has a probability $p_i = 1 - e^{-\Delta t \cdot \lambda_i(t)}$ of becoming infected. The probability of becoming infected depends on the instantaneous risk of infection $\lambda_i(t)$, computed at any time of the simulation. The risk of infection for each individual is defined as the sum of the risk factors coming from the different sources of infections considered, namely:

1. contacts with infectious members of the household (first term in Eq. 5),
2. contacts with infectious individuals attending the same school (second term in Eq. 5),
3. contacts with infectious work colleagues (third term in Eq. 5),
4. random contacts in the population and cross-border diffusion (fourth term in Eq. 5),
5. long distance travel (fifth term in Eq. 5).

$$
\begin{aligned}
\lambda_i &= \sum_{\left\{k=1,\ldots,N_{S_i}|H_k=H_i\right\}} \frac{I_k\beta_h}{n_i} \\
&+ \sum_{\left\{k=1,\ldots,N_{S_i}|P_k=P_i\right\}} \frac{I_k\beta_s}{m_i} \\
&+ \sum_{\left\{k=1,\ldots,N_{S_i}|P_k=P_i\right\}} \frac{I_k\beta_w}{m_i} \\
&+ \sum_{\left\{k=1,\ldots,N|S_k\in S_i^\star\right\}} \frac{I_k\beta_c f(d_{ik})}{\sum_{\left\{k=1,\ldots,N|S_k\in S_i^\star\right\}} f(d_{ik})} \\
&+ \sum_{\{k=1,\ldots,N|S_k\neq S_i\}} \frac{I_k\beta_t P(S_i,S_k)}{N_{S_k}} \qquad (5)
\end{aligned}
$$

The terms in Eq. (5) are defined as follows:

- S_i is the index of the country where individual i lives in.
- H_i is the index of the household where individual i lives in and n_i is the household size.
- P_i is the index of the school/workplace where individual i studies/works (depending on the employment of i) and m_i is the school/workplace size.
- N is the European population.
- N_{S_k} is the population of country S_k.
- $S_i^\star$ is the set indices of the countries bordering country S_i, country S_i included. This set of countries ($S_i^\star$) allows taking into account random contacts in the general community in country S_i and cross-borders diffusion due to random contacts among individuals living in bordering countries ($S_i^\star \setminus S_i$).
- $I_k = 1$ if individual k is infected, 0 otherwise.
- $f(d_{ik}) = (d_{ik} + r_g^0)^{-\beta_r} \exp\left\{-\frac{d_{ik}}{\kappa}\right\}$, where $r_g^0 = 5.8$km, $\beta_r = 1.65$ and $\kappa = 350$km, makes the transmission of the epidemic in the general community (and the cross-border diffusion) explicitly dependent on patterns of human mobility (as described in González et al. (2008)).
- $P(S_i, S_k)$ is the probability of travelling from country S_i to country S_k. It is estimated by using the gravity model proposed in (Merler and Ajelli, 2010), namely $F_{ik} = \theta \frac{g_k^{\tau_t} g_i^{\tau_f}}{d_{ik}^{\rho}}$, where g_i is a normalized GDP of country i ($g_i = \frac{G_i}{G^\star} p_i$ where G_i is the GDP per capita of country i, $G^\star$ is the average GDP per capita of EU27 and p_i is the population of country i) and d_{ik} is the distance between the two countries. τ_f and τ_t tune the dependence of dispersal on donor and recipient sizes, ρ tunes the dependence on the distance and θ is

a proportionality constant. Best estimates of the parameters of the gravity model are: $\tau_t = 0.9860282$, $\tau_f = 0.5688141$ and $\rho = 0.3895469$.

- β_h (expressed in day^{-1}) is the within–household transmission rate.
- β_s (in day^{-1}) is the within–school transmission rate.
- β_w (in day^{-1}) is the within–workplace transmission rate.
- β_c (in day^{-1}) is the transmission rate in the general community.
- β_t (in day^{-1}) is the transmission rate for long-distance travel.

At any time t of the simulation, infected (exposed) individuals enter the infectious phase at the rate $\Delta t/T_L$, where T_L is the latent period. Finally, infectious individual recover from the infection at the rate $\Delta t/T_I$, where T_I is the infectious period.

1.4 Natural history of influenza and model parametrization

The epidemiological flow of influenza can be schematized as follow. A newly infected person passes through latent and infectious phases. After the infectious phase, either individuals recover and acquire (partial or complete) immunity, or (extremely rarely for H1N1pdm infections) die.

Symptomatic and asymptomatic cases In recent studies (e.g. Merler and Ajelli (2010); Chao et al. (2010); Ciofi degli Atti et al. (2008); Gojovic et al. (2009); Ferguson et al. (2006); Longini et al. (2004); Halloran et al. (2008)), the probability of developing severe symptoms and augmented viral load in symptomatic cases have been taken into account. However, given the lack of reliable data on these parameters for the H1N1pdm, we did not distinguish between symptomatic and asymptomatic individuals in the model.

Induced mortality, multiple strains, behavioural changes Our model is kept very simple in several other aspects. Induced mortality is not considered (Longini et al., 2005; Ciofi degli Atti et al., 2008; Ferguson et al., 2006; Germann et al., 2006). Multiple strains (Chowell et al., 2006) or the contemporary presence of other diseases potentially affecting the influenza dynamics (Merler et al., 2008) are not considered. Spontaneous changes in population behaviour in response to a pandemic and potentially affecting its course (Ferguson, 2007; Poletti et al., 2009) are neglected.

Differential susceptibility to infection We assume reduced susceptibility for older age classes, consistent with the early case data available from Mexico (Fraser et al., 2009) and the UK (Ghani et al., 2009) and broadly consistent with the later observation of significant pre-pandemic antibody titres to H1N1pdm in a fraction of older individuals (Zimmer and Burke, 2009; Xing and Cardona, 2009; Miller et al., 2010). As baseline value, we assume that children are more susceptible (by a factor 2) to the infection than adults (Fraser et al., 2009). We also explore values of 1 (no differential susceptibility to the infection) and 4.

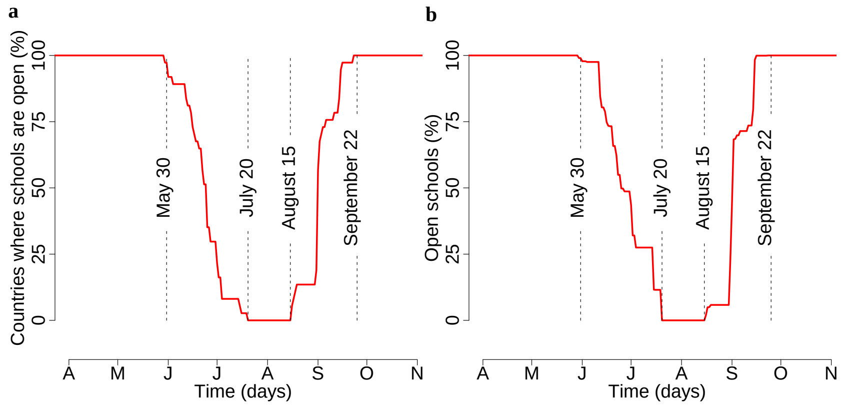

Figure S6: **a** Percentage of countries where schools are open over time, according to the 2009 scholastic calendars. **b** Percentage of open schools in Europe, computed as in **a** but by weighting the countries by their population size.

Seasonality / School holidays The mechanism responsible for the seasonality of influenza epidemics are still debated (Lipsitch and Viboud, 2009). In particular, recent studies have pointed out how the vapour pressure could be a driving factor for this process (Shaman and Kohn, 2009; Shaman et al., 2010). A brief analysis of whether vapour pressure could explain the observed pattern of spread has been carried out in Sec. 2 without clearcut results. Therefore, in each simulated epidemic the transmission rates are kept constant, but for the summer and autumn school holidays. In fact, we explicitly considered the closure of schools for the summer and autumn holidays (European Commission, 2008) (see Tab. S1), giving rise to a seasonal effect as shown in Fig. S6. As for the summer holidays, schools were closed in almost the whole Europe from mid July to the beginning of September and this can have played a relevant role in the spread of H1N1pdm influenza because of the high transmissibility of H1N1pdm influenza in schools (Ghani et al., 2009). The role of schools in influenza epidemics is quantified in Cauchemez et al. (2008) where the authors also predict the potential effect of school closure during a pandemic. During holidays, we assume that the transmission parameter in the general community (β_c, see Eq. (5)) increases by a factor 1.4 (Cauchemez et al., 2008)). We found that model predictions are not very sensitive to the value of this parameter (see Fig. S7).

Sickness-induced absenteeism As most transmissions of H1N1pdm virus have been found to occur soon before or after the onset of symptoms in patients (Ghani et al., 2009; Cauchemez et al., 2009), it is hardly likely that absenteeism has remarkably reduced transmission in schools. Thus, we did not consider sickness-induced absenteeism in the model.

Generation time In early studies on the transmission dynamics of H1N1pdm influenza in UK (Ghani et al., 2009), the average generation time T_g (the time between symptom onset in an individual and the moment of symptom onset in her/his presumed infector) has been estimated to 2.6 days. Most other estimates are similar (Fraser et al., 2009; White et al., 2009; Donnelly et al., 2011), both for H1N1pdm and for previous pandemics, although much

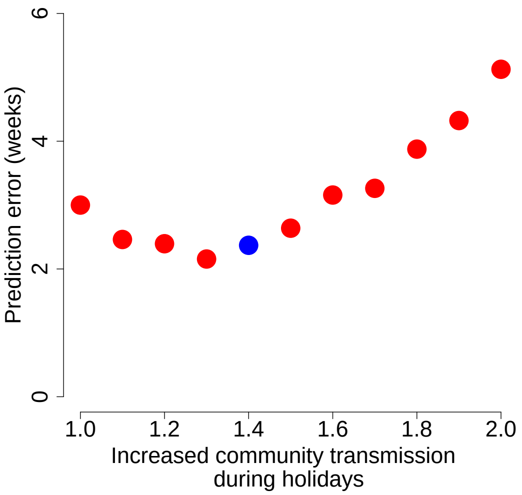

Figure S7: Prediction error (average of the absolute value of observed minus predicted autumn peak) of the baseline simulation ($R_0^{UK} = 1.48, T_g = 3.1$ days) as a function of the increased community transmission during holidays. The blue point, indicating an increase of about 40% of the transmission rate in the general community during holidays (Cauchemez et al., 2008), represents the value used in all simulations.

longer estimates have appeared (Cowling et al., 2009; Yang et al., 2009).

Obtaining reliable estimates for the latent period (the time between infection and the beginning of the infectious period) is instead more difficult. On the basis of results from experimental infections, Ferguson et al. (2005) had used a latent period of 1.5 days, and we keep to that estimate. As baseline we used an infectious period of 1.6 days, giving a generation time of 3.1 days, but this has been extensively varied between 2.1 and 3.7 days, covering most reasonable estimates.

For the sake of simplicity, we assume that both latent and infectious periods are exponentially distributed (as in classical mathematical models of infectious diseases). We acknowledge that this assumption is fairly unrealistic, but overall the distribution of infectiousness over the time since infection is not so different from the empirical distribution shown in (Ghani et al., 2009). A more realistic distribution of the generation time is assumed in (Ferguson et al., 2006), where individuals are assumed to transmit more at the very beginning of the infectious period, giving rise to faster simulated epidemics and to higher peak daily attack rates. As shown in (Merler and Ajelli, 2010), while these different modelling choices can affect the evaluation of some containment strategies (e.g. antiviral treatment and prophylaxis), they do not affect, as long as R_0 and T_g are kept the same, the spread of uncontrolled epidemics, as it is the case of this work.

R_0 R_0 depends not only on the transmissibility characteristic of the virus, but also it greatly depends on the structure of the host population. Therefore, it is not possible to set a unique R_0 for the whole study area without changing the values of the transmission rates in the different

Label	Country	Summer holidays		Autumn holidays	
		From	To	From	To
AL	Albania⋆	25/6	31/8	–	–
AD	Andorra†	23/6	15/9	–	–
AT	Austria	4/7	5/9	27/10	31/10
BE	Belgium	1/7	31/8	2/11	6/11
BG	Bulgaria	15/6	15/9	5/11	9/11
BA	Bosnia-Herzegovina⋆	25/6	31/8	26/10	1/11
CH	Switzerland	17/6	3/9	–	–
CZ	Czech Republic	1/7	31/8	29/10	30/10
DE	Germany	15/7	28/8	12/10	24/10
DK	Denmark	27/6	15/8	10/10	18/10
ES	Spain	23/6	15/9	–	–
EE	Estonia	4/6	31/8	24/10	1/11
FI	Finland	30/5	15/8	29/10	1/11
FR	France	2/7	1/9	24/10	5/11
UK	United Kingdom	20/7	7/9	26/10	30/10
GR	Greece	16/6	10/9	–	–
HR	Croatia⋆	25/6	31/8	26/10	1/11
HU	Hungary	16/6	31/8	23/10	1/11
IE	Ireland	30/6	5/9	26/10	30/10
IT	Italy	10/6	15/9	–	–
LI	Liechtenstein	4/7	17/8	5/10	18/10
LT	Lithuania	1/6	31/8	26/10	1/11
LU	Luxembourg	16/6	14/9	31/10	8/11
LV	Latvia	1/6	31/8	26/10	30/10
MC	Monaco #	2/7	1/9	24/10	5/11
MK	Macedonia⋆	25/6	31/8	26/10	1/11
MT	Malta	27/6	22/9	2/11	3/11
NL	Netherlands	11/7	23/8	17/10	25/10
NO	Norway	21/6	17/8	28/9	5/10
PL	Poland	20/6	31/8	–	–
PT	Portugal	22/6	13/9	–	–
RO	Romania	13/6	13/9	2/11	8/11
RS	Republic of Serbia⋆	25/6	31/8	26/10	1/11
SK	Slovakia	1/7	31/8	29/10	30/10
SI	Slovenia	25/6	31/8	26/10	1/11
SE	Sweden	12/6	31/8	26/10	1/11
CY	Cyprus	18/6	2/9	–	–

⋆ assumed as in Slovenia
† assumed as in Spain
# assumed as in France

Table S1: Summer and autumn holidays in the primary schools of the capital city of the different European countries.

countries. Since there are neither evidence nor reliable reasons for assuming differences among the European countries in the probability of transmitting the infection in the different social contexts, we fixed the transmission rates to obtain a certain reproduction number in UK, R_0^{UK}, compliant with observed data (see below), and then we used the same transmission rates in all countries of the study area. As a consequence of the different demographic structures, R_0 is highly variable from country to country (see Fig. S8). R_0 is negatively correlated to the average age of the population (Spearman's correlation coefficient $r = -0.37$, $p = 0.03$). As a consequence of the larger susceptibility to infection of children with respect to adults, R_0 is more strongly correlated to the fraction of individuals aged < 15 years old (Spearman's correlation coefficient $r = 0.58$, $p < 0.001$). Details on the computation of the basic reproductive number are given in Sec. 1.5.

In order to choose sensible values for R_0^{UK}, we look at the early cases in UK. In the main

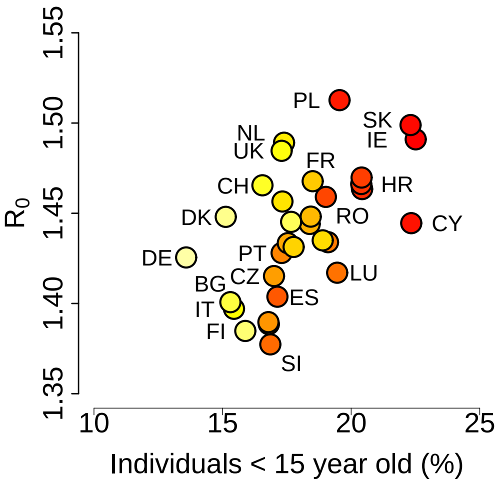

Figure S8: Country-specific values of R_0 in the baseline simulations ($R_0^{UK} = 1.48, T_g = 3.1$ days). Values of R_0 are plotted against the fraction of individuals < 15 year old in the different European countries. Colours represent the average age of the population, decreasing from yellow (42 years) to red (32.5 years).

text we show the results of the analysis of QSurveillance data. Here we show the results of the analysis of HPA case estimates. By fitting an exponential model to the UK data up to July 5, one get estimates for the exponential growth rate r between 0.14 and 0.175 days^{-1}, corresponding to a doubling time between 4 and 5 days, which would give R_0^{UK} between 1.41 and 1.53 (by assuming a generation time T_g of 2.7 days) , while a longer T_g (3.1 days) would imply R_0^{UK} between 1.48 and 1.62. Simulations with $R_0^{UK} = 1.48$ and $T_g = 3.1$ days give rise to doubling times of 5 days (95%CI 4.3–5.5) in UK, while simulations with $T_g = 2.7$ days give rise to doubling times of 4.8 days (95%CI 4.2–5.3) in UK. Therefore, $R_0^{UK} = 1.48$ (in UK) and $T_g \in [2.7, 3.1]$ days can be considered central estimates. However, we varied extensively T_g (between 2.1 and 3.7 days), see Fig. 4A in the main text.

Estimates of R_0^{UK} were obtained by fitting an exponential model $i(t) \approx a + be^{rt}$ to the weekly number of cases to obtain an estimate of the growth rate of the epidemic to be inserted in Eq. (4). The same procedure was applied to obtain estimates of R_0^{UK} from QSurveillance data shown in the main text. This is also the procedure used for estimating R_0^{UK} in the simulations (see Sec. 1.5).

Proportion of transmission in the different social contexts According to available data on the proportion of transmission in different social contexts for seasonal influenza (Ferguson et al., 2005, 2006; Cauchemez et al., 2008), the transmission model is parametrized so that in UK 18% of transmission occurs through contacts made at school, 30% within households, 19% in workplaces and 33% in the general community. As stated above, we assume age-dependent susceptibility to infection. Specifically, in the baseline simulations we assume susceptibility to infection of children relative to adults equal to 2 (Fraser et al., 2009;

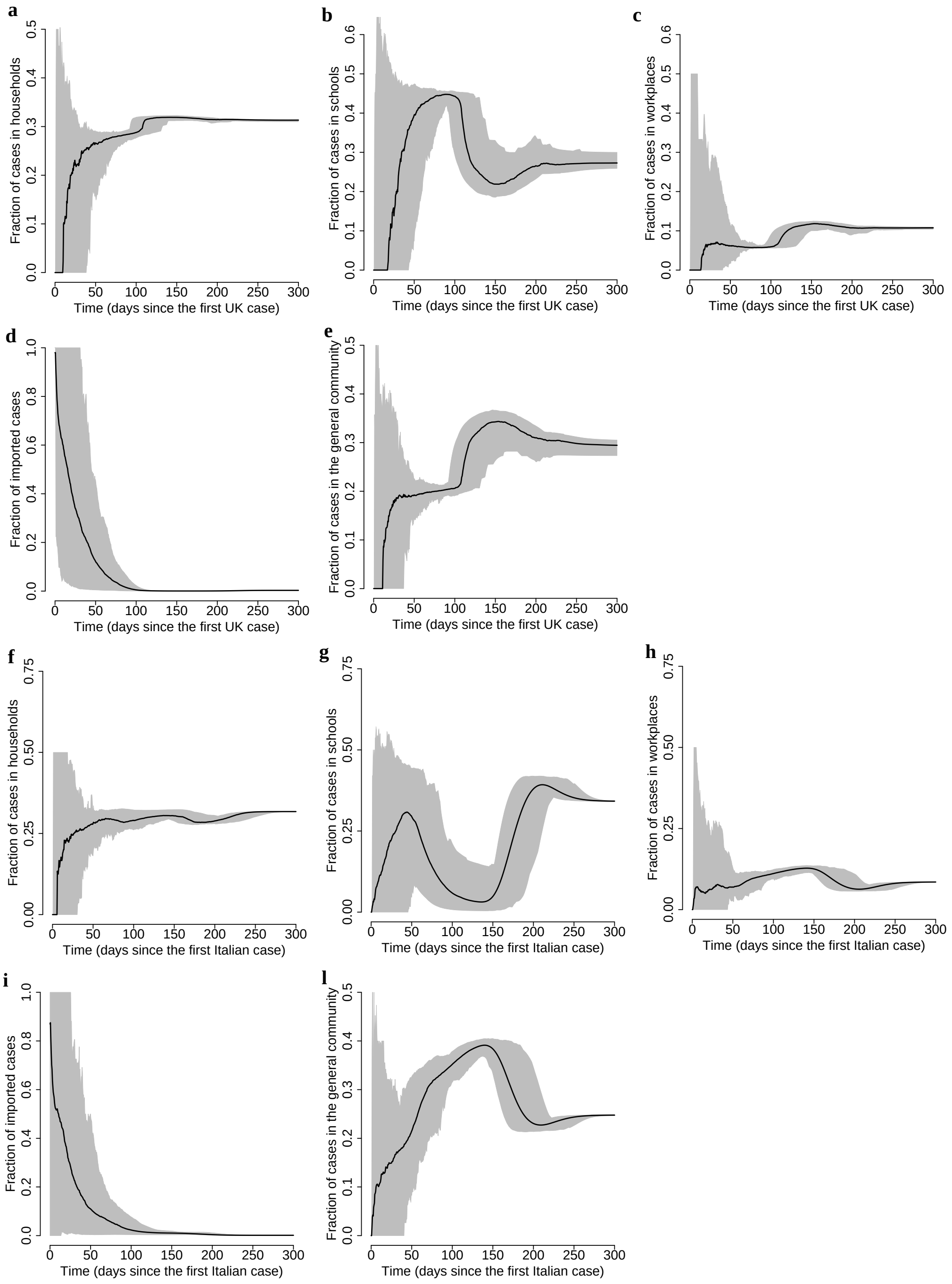

Figure S9: Cumulative fraction of cases over time generated within households (**a**), in UK. The solid dark lines represent the median value, the shaded areas represent 95%CI. **b** As **a** but for cases generated within schools. **c** As **a** but for cases generated within workplaces. **d** As **a** but for imported cases. **e** As **a** but for cases generated in the general community. **f**,**g**,**h**,**i**,**l** As **a**,**b**,**c**,**d**,**e** but for Italy.

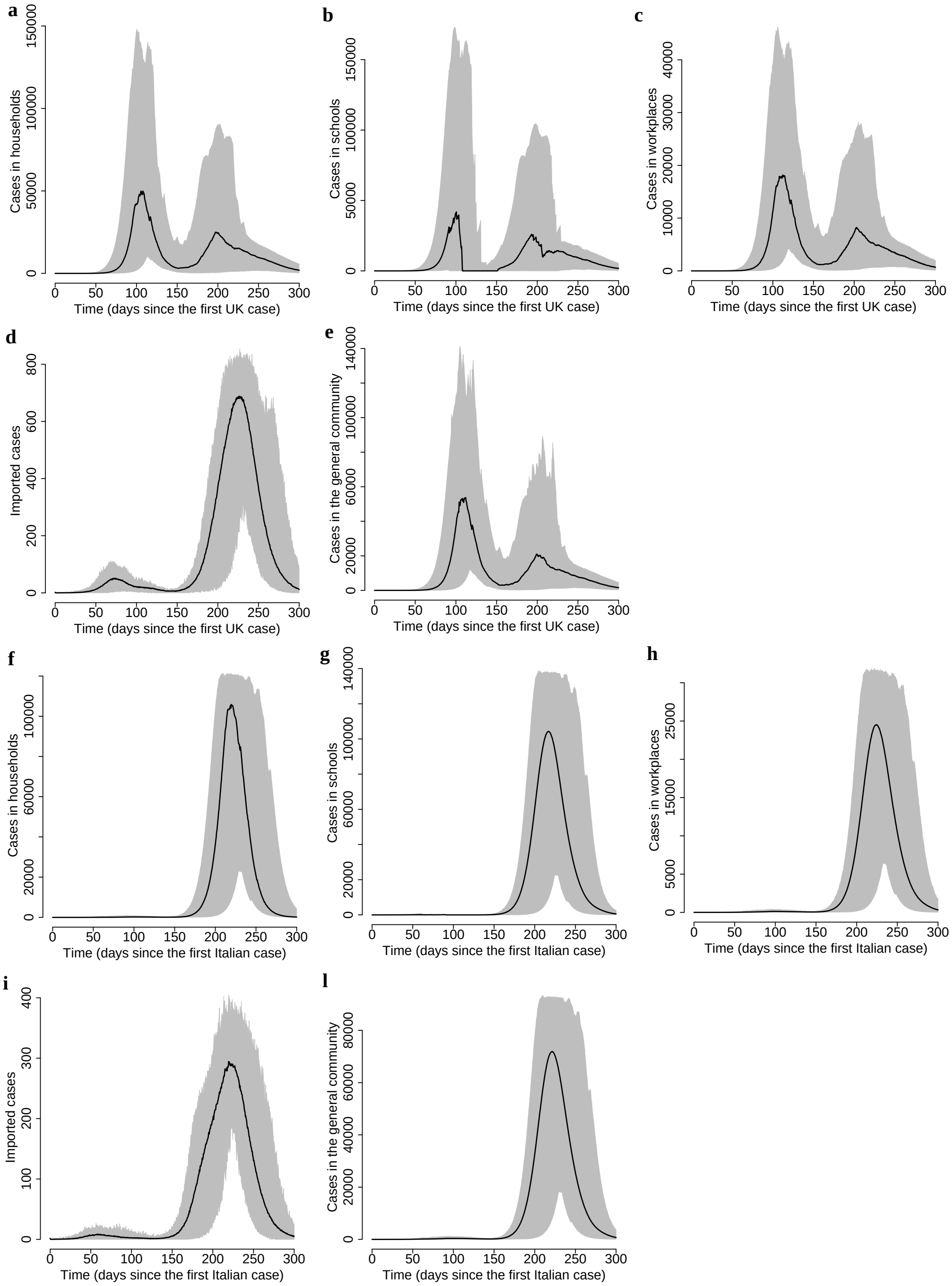

Figure S10: Number of daily cases over time generated within households (**a**), in UK. The solid dark lines represent the median value, the shaded areas represent 95%CI. **b** As **a** but for cases generated within schools. **c** As **a** but for cases generated within workplaces. **d** As **a** but for imported cases. **e** As **a** but for cases generated in the general community. **f**,**g**,**h**,**i**,**l** As **a**,**b**,**c**,**d**,**e** but for Italy.

Cauchemez et al., 2009). As a consequence of the age-dependent susceptibility to infection, the figure becomes 36% of cases in schools, 31% in households, 9% in workplaces and 24% in the general community.

After having parametrized the model in UK (i.e., by simulating an epidemic spreading in UK without considering connections with the rest of Europe), the same transmission rates are assumed in the rest of the study area. As the contact rates are determined by the sociodemographic structure of the population, we are somehow setting the probability of transmitting the infection in the different social contexts. The fraction of cases in the different social contexts, as predicted by the model for UK during the epidemic course, is shown in Fig. S9**a-e** (Fig. S10**a-e** reports the daily number of cases in the different social contexts). Predictions are in good agreement with observed data at the very beginning of the epidemic (Ghani et al., 2009). Afterward, the effect of the closure of schools for the summer holidays is clearly observable. Unsurprisingly, the fraction of cases infected abroad decreases down to 0 in the long-term, and the fraction of infections in other social contexts increases correspondingly. Unlike UK, the epidemic in the rest of Europe was basically absent during the summer, as predicted by the model. As an example, in Italy we found that the fraction of imported cases was very high during the whole summer (see Fig. S9i and see Fig. S10i) with very low transmission in schools, households, workplaces and the general community even before the summer holidays (see Fig. S9fghl and Fig. S10fghl), in good agreement with observed data. In fact, the analysis of the first 152 laboratory-confirmed cases of H1N1pdm influenza in Italy revealed that 86% of them believed to have been infected abroad, primarily either in US or in Mexico (Rizzo et al., 2009).

Long–distance travel We chose parameter β_t on the basis of the following criteria: the total number of nights spent in 2007 by EU27 citizens in collective tourist accommodation establishments in EU27 countries outside their own member state is 974,114,000 (Eurostat, 2009), that is 0.4794 % of the days of the year. Thus, we set β_t so that the number of cases generated abroad is about the 0.4794 % of the overall number of cases.

Setting the time in the simulations According to the WHO daily reports (World Health Organization, 2009), 46 laboratory confirmed cases were reported in European countries from 27 April 2007 to 3 May 2009 (1 in Austria, 1 in Denmark, 2 in France, 6 in Germany, 1 in Ireland, 1 in Netherlands, 13 in Spain, 1 in Switzerland and 15 in UK). In the simulations, cases are imported through the procedure described in Sec. 1.2. We set the time in the simulations to 3 May 2009 as soon as the cumulative number of (simulated) imported cases is ≥ 46. This gives a reliable way for setting the time in the simulations.

1.5 Computation of R_0

For compartmental epidemic models, R_0 is defined as the dominant eigenvalue of the next–generation operator of the model (Diekmann et al., 1990). For more structured models, e.g. accounting for households and other kind of “small” groups (e.g., schools, classes, workplaces, etc.), the same definition of R_0 is uncertain (Goldstein et al., 2009). Therefore, as in (Ciofi

degli Atti et al., 2008; Ferguson et al., 2006; Germann et al., 2006; Ajelli and Merler, 2008; Merler and Ajelli, 2010), we compute R_0 of the simulated epidemic (actually R_0^{UK}, the reproduction number of simulations for UK) from the intrinsic growth rate of the simulated epidemics, using Eq. (4) discussed above. The growth rate refers to the natural increase of the number of infected individuals during the initial phase of the epidemic, when the effects of susceptible depletion are negligible (and intervention strategies are not considered).

The intrinsic growth rate r was estimated by considering an epidemic spreading in UK only (without considering imported cases). In order to (partially) eliminate the stochastic effects in the initial phase of the epidemic, we initialized the simulation with 100 infected individuals randomly chosen in the population. The intrinsic growth rate r and the resulting value of R_0^{UK} were obtained by fitting an exponential model $i(t) \approx a + be^{rt}$ to the daily number of cases (during the initial phase of the simulated epidemic, lasting 1 month). Finally, R_0^{UK} was computed by averaging over 100 experiments.

One could reasonably argue that for this kind of models (where individuals are explicitly represented) R_0^{UK} could be estimated directly by the number of cases generated from the index cases at the beginning of the epidemic (as in Germann et al. (2006); Ajelli and Merler (2008)), instead of using Eq. (4) (since that equation is derived for homogeneous mixing and the population in our model does not mix homogeneously). However, averaging correctly over the different types of individuals and settings of infections is not obvious (Diekmann et al., 1990).

1.6 Realizations and resources

Each result presented in the paper is based on 100 realizations of the same experiment. This certainly represents a number large enough to guarantee the stability of the results. Specifically, only the timing and the geographic location of the initial cases are highly variable (however this is due to the high stochasticity of the epidemic in its initial phase and not to the number of simulations considered). On the contrary, the epidemiological indicators depending on the whole course of the epidemic (e.g., the final attack rate) are very stable (but for very small countries). The model was implemented in C language. For running a simulation on the European population (accounting for about 515 millions of individuals explicitly represented in the model) the amount of RAM memory required is about 9 GB. A simulation takes about 40 minutes on a single CPU of an Intel Xeon 2.4-GHz.

2 Climatic factors

We also considered the possibility that explicit climatic effects could explain the difference between observed data and model predictions. However, we did not find a significant correlation between observed peak week and the minimal daily temperature[1] (average value over the country and over September 2009) in the different countries (see Fig. S11). The same holds by considering absolute humidity (vapour pressure), longer time intervals or the difference between observed and predicted peak week. Vapour pressure is calculated, under the

[1] Data are available at `http://eca.knmi.nl/download/ensembles/ensembles.php`, (Haylock et al., 2008).

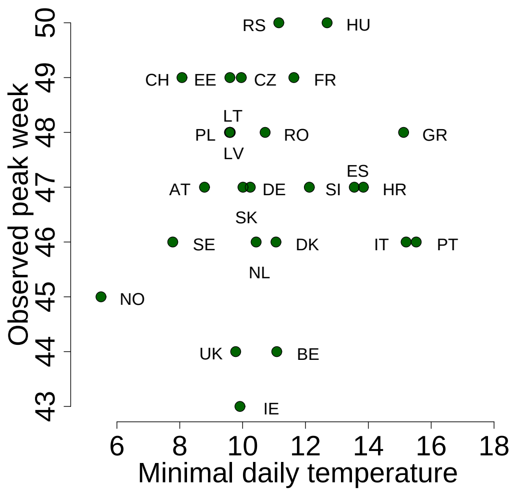

Figure S11: Minimal daily temperature (average over the country and over September 2009) plotted versus observed autumn peak week.

hypothesis that dew-temperature remains constant over the day (a good approximation for much of Europe (Anderson-Sköld et al., 2008)), using the Clausius-Clapeyron relation:

$$e_s(T_d) = e_s(T_0)\exp\left\{\frac{L}{R_v}\left(\frac{1}{T_0} - \frac{1}{T_d}\right)\right\} \ ,$$

where T_d is the dew-point temperature, $e_s(T_0) = 6.11$mb is the vapour pressure at the reference temperature $T_0 = 273.15$K, L is the latent heat of evaporation of water and R_v is the gas constant for water vapour. According to (Anderson-Sköld et al., 2008), we approximated the dew-point temperature with the daily minimum temperature, which is equivalent to saying that relative humidity is approximately 100% over the night (a quite good assumption in a wide range of climates, Europe included).

3 Predictions of homogeneous mixing models

We also considered two simple stochastic (discrete–time, time step 0.5 days, as in the individual based model) homogeneous mixing SEIR models. The main characteristics of the first model are:

- the infection is seeded in the different countries by using data on importation of cases from US and Mexico (as described in Sec. 1.2 for the individual based model);
- the epidemic spread in each country is modelled without considering fluxes of travellers from/to other European countries;
- we consider an infectivity period of 1.6 days and a latent period of 1.5 days, as in the baseline simulations of the individual based model;

- $R_0 = 1.48$ is assumed when schools are open, as in the individual based model; in this model, however, R_0 is the same in all countries because of the lack of demographic heterogeneity;

- in each country the school calendar determines different transmission levels over time. During holidays R_0 switches to a lower vale ($R_0^s < R_0$);

- we optimized the value of R_0^s during holidays in order to fit the observed autumn peak week.

Basically, this model can be simulated by using Eq. (5) with $\beta_h = 0$, $\beta_s = 0$, $\beta_w = 0$, $\beta_c = R_0\gamma = 1.48/1.6$, where γ^{-1} is the length of the infectious period, $\beta_t = 0$, $f(d) \equiv 1$, and excluding cross-border diffusion.

The second model we considered is similar to the model described above, but fluxes of travellers from/to other European countries are explicitly considered (same as long–distance travel in the individual based model). This model can be simulated as the previous one after having estimated β_t so that the number of cases generated abroad is about the 0.4794 % of the overall number of cases, as in the individual based model (see Sec. 1.4 paragraph Long–distance travel).

As shown in Fig. 7 in the main text, both models are able to predict the autumn peak week. However, some limitations should be considered: firstly, predictions are very sensitive to the value of R_0^s during the holidays, and its optimal value was difficult to predict before the end of the epidemic, thus making it very difficult to use these models for real–time prediction. In addition, as shown in Fig. 7B in the main text, the optimal reduction of R_0 during holidays is different from the values suggested in the literature (Hens et al., 2009). Also, note that the optimal reduction for the model not accounting for long–distance travel across European countries is lower than that for the model accounting for long–distance travel. This can be explained by considering that in the simpler model a higher transmission during the summer is required to fit the autumn peak week in the different countries (especially in the Eastern part of Europe), where the infection is seeded with imports from US and Mexico only. Moreover, both models fail to predict the dynamics of the epidemic during the summer in UK (only just one single summer wave is predicted by these models in UK) and in other European countries (for instance, a relevant summer wave is consistently predicted in Germany, probably because of the lack of demographic structure: in fact, Germany has one of the oldest populations in Europe). Remarkably, by assuming $R_0^s \in [0.8R_0, 0.9R_0]$, i.e. values suggested in (Hens et al., 2009), the prediction error is about 8 weeks, which implies a single summer wave in almost all European countries. Finally, both models predict cumulative attack rates of about 57% (except for countries, like UK, France and Germany, where the mitigating effects of school closure is relevant) which seems quite unrealistic.

4 Sensitivity analysis

4.1 Age-dependent susceptibility to infection

4.1.1 Susceptibility of children relative to adults: 4/1

In the main text we assume age-dependent susceptibility to infection. Specifically, we assume that the susceptibility to infection of children (individuals aged < 15 years old) relative to adults (individuals aged ≥ 15 years old) is 2. This results in very large attack rates in younger age groups with respect to adults, compliant with observed data (Miller et al., 2010). Here we assume that the susceptibility to infection of children relative to adults is 4 (the transmission rates were re-estimated in order to give $R_0^{UK} = 1.48$ and to reproduce the proportion of cases in the different social contexts, as in the baseline simulations shown in the main text). Results are shown in Fig. 6 in the main text. The difference in the attack rates by age is remarkable (ranging from about 55% in individuals aged less than 15 to about 10% in adults). However, the reduced transmissibility in adults results in a slightly lower probability of observing a summer wave. More remarkably, a delay in predicting the autumn peak week was observed. This is due to a more remarkable decrease of the transmission in the decay phase of the epidemic during summer holidays, resulting in a much lower depletion of susceptible individuals (see Fig. S12a-d). In fact, the timing of simulated epidemics with different age-dependent susceptibility to infection does not change much if we do not consider summer holidays (see Fig. S12e-f). The mean deviation between observed and predicted peek week is on average 6 weeks, while the mean deviation between observed peek week and peek week as predicted by the baseline simulations is on average 2.2 weeks (results shown in the main text). These results suggest that the susceptibility to infection of children relative to adults is likely to have been less than 4.

4.1.2 Susceptibility of children relative to adults: 1/1

In the main text, the transmission model is parametrized so that in UK 18% of transmission occurs through contacts at school, 30% within the households, 19% through contacts in workplaces and 33% in the general community. Moreover, we assume that the susceptibility to infection of children relative to adults is 2. As a consequence, the figure becomes 36% of transmission occurs through contacts made at school, 31% within the households, 9% through contacts in workplaces and 24% in the general community. These data were available in May-June 2009. Fig. 6 in the main text shows that using only data available prior this date (e.g., without assuming age-dependent susceptibility to infection; the transmission rates were re-estimated in order to give $R_0^{UK} = 1.48$ and to reproduce the proportion of cases in the different social contexts, as in the baseline simulations shown in the main text) would have led to overestimate the attack rate as a consequence of the larger fraction of cases in adults and elderly age groups, which in turn depends on the larger fraction of cases generated in the general community. This is also the reason why predicting the impact of school holidays on the timing of the autumn wave would have been more difficult a priori (see Fig. S12 and discussion above on the effects of summer holidays on the epidemic timing). The simulated epidemics spread much faster than observed in autumn (the mean deviation between observed

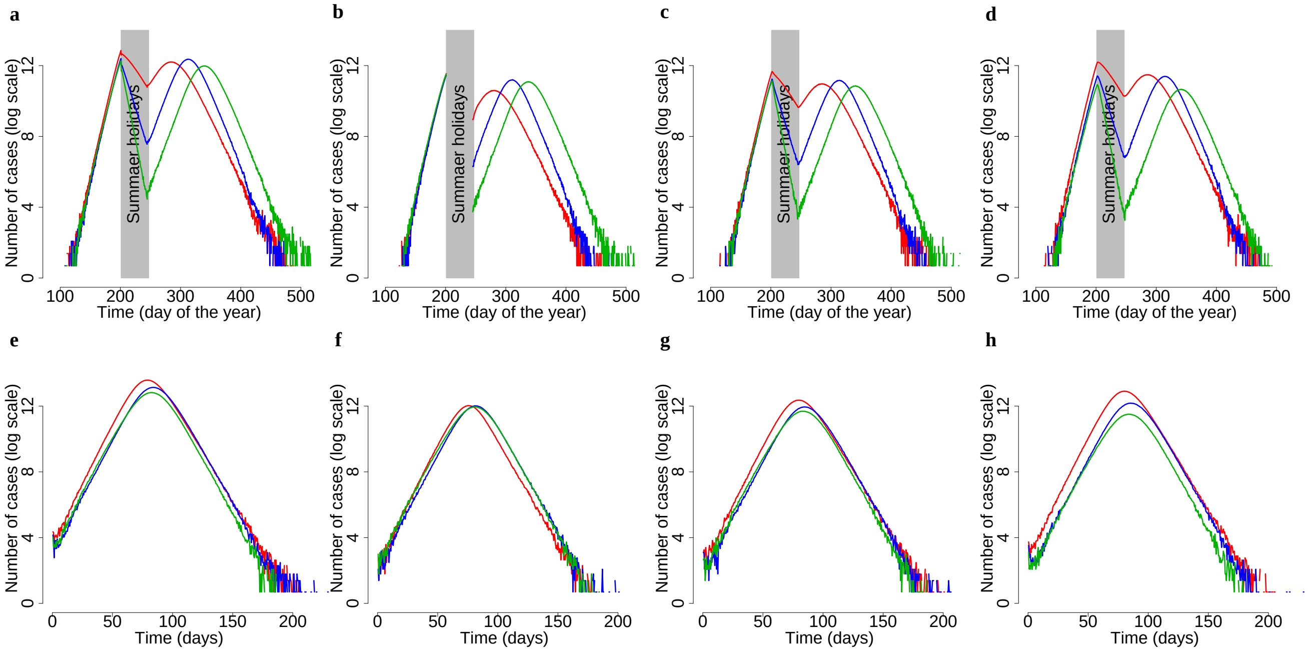

Figure S12: **a** Total number of daily cases in UK as obtained by assuming $R_0^{UK} = 1.48$, $T_g = 3.1$ days and susceptibility to infection of children relative to adults equal to 1 (red line), equal to 2 (blue line, as in the baseline simulations shown in the main text) and equal to 4 (green line). For each scenario, the result of one simulation as obtained by running the European model is shown. **b** As **a** but for the number of daily cases in schools. **c** As **a** but for the number of daily cases in households. **d** As **a** but for the number of daily cases in the general community. **e**, **f**, **g** and **h** As **a**, **b**, **c** and **d** but for simulations as obtained by running the model only in UK, without considering summer holidays, initialized with 100 initial infected individuals at time 0 to reduce the influence of the initial stochasticity on the epidemic timing.

and predicted peek week is 8 weeks on average), and the probability of observing a summer wave quite relevant in size in some Western countries (notably Ireland, the Netherlands and France) would have been remarkable.

4.2 Sociodemographic structure

The role of population heterogeneity in the spread of influenza pandemic has been analyzed in (Merler and Ajelli, 2010). Here we show the implications in terms of timing and impact on the spread of H1N1pdm influenza. To such aim we assume the same epidemiological parameters (infective period = 1.6 days, latent period = 1.5 days and susceptibility to infection of children relative to adults = 2) and the same transmission parameters in the different social contexts used in the main text and we change the sociodemographic structure of the different countries. Specifically, we considered 2 different scenarios, namely:

- scenario a) we assume the sociodemographic structure of Ireland (the youngest European

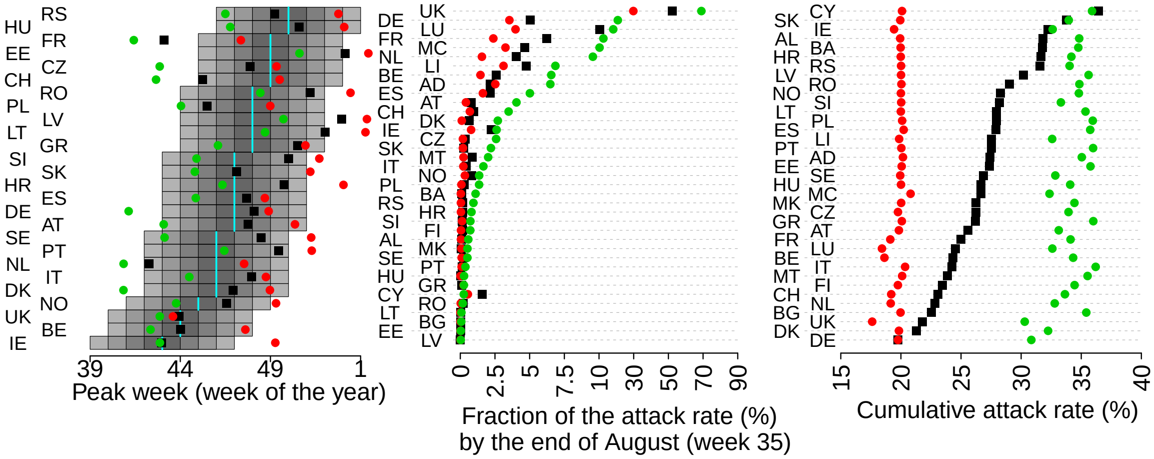

Figure S13: **a** Observed peak week (vertical cyan bars) for European countries covered by the WHO/Europe weekly influenza surveillance system plotted versus peak week as predicted in the baseline simulations (black squares), as predicted by assuming the sociodemography of Ireland in all countries (green circles), as predicted by assuming the sociodemography of Germany in all countries (red circles) ; only the Autumn wave is considered for UK. **b** Activity up to week 35 in the three scenarios considered (colours as in **a**). **c** Cumulative attack rate in the three scenarios considered (colours as in **a**).

country) in all European countries,

- scenario b) we assume the sociodemographic structure of Germany (the oldest European country) in all European countries.

Therefore, in these two scenarios, the populations of European countries differ only by the total number of individuals (and, consequently, for the population density), while the sociodemographic structure (e.g., age-structure, household size and composition, fraction of students and fraction of workers in the population) is kept constant.

Fig. S13 shows that, unsurprisingly, scenario a) gives rise to faster epidemics and larger cumulative attack rates, as an effect of the change of R_0 in the different countries (which is now uniform across countries). In fact, in scenario a) R_0 is approximately 1.49 in all countries (i.e. the value of R_0 in Ireland in the baseline simulations, see Fig. S8) while in scenario b) R_0 is approximately 1.43 in all countries (i.e. the value of R_0 in Germany in the baseline simulations, see Fig. S8). Fig. S13 shows that the increase of R_0 in scenario a) (induced by the change of the sociodemographic structure, we stress the fact that we did not change any transmission parameters) results in a remarkable increase of the probability of observing a summer wave in UK and also in other Western countries, like Germany and the Netherlands and the epidemic spreads much faster in Europe in autumn. The opposite pattern is observed in scenario b).

As expected, in scenario a) the same cumulative attack rate as predicted by the baseline simulations for Ireland is obtained in all countries (about 35%). The larger variability observed

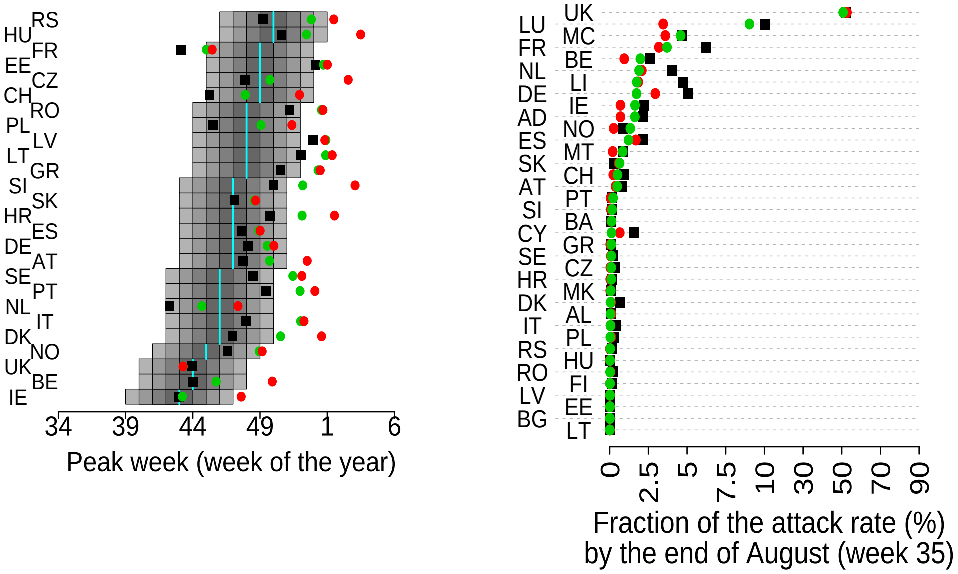

Figure S14: **a** Observed peak week (vertical cyan bars) for European countries covered by the WHO/Europe weekly influenza surveillance system plotted versus peak week as predicted in the baseline simulations (black squares), as predicted by not considering long–distance travels between European countries (green circles), as predicted by not considering both long–distance travels and cross-border–diffusion (red circles) ; only the Autumn wave is considered for UK. **b** Activity up to week 35 in the three scenarios considered (colours as in **a**).

in some countries depends on the size of the summer wave. Qualitatively similar results are obtained by considering scenario b) (attack rate about 20% io all countries, similar to attack rate predicted for Germany in the baseline simulations).

These results suggest that the sociodemographic structure not only affects the epidemic impact in the different countries but also contributes to determine the timing (together with the number of imported cases from US and Mexico and across the different European countries, and timing of school holidays). This provides a further support to the need of including heterogeneity in the sociodemographic structure for capturing epidemic spread.

4.3 Patters of human mobility

4.3.1 Inter–European travel

Fig. S14 shows the effects of not considering long–distance travel across European countries and of not considering neither long–distance travel nor cross–border diffusion. Over the summer, the simulated epidemics do not differ much from the baseline simulations shown in the main text, while remarkable differences are observed in autumn in those countries less connected with US and Mexico. In fact, the epidemic timing in those countries with strong connections with US and Mexico (Ireland, UK, Germany, the Netherlands, France) is strongly determined by timing and number of imported cases from US and Mexico. Quite

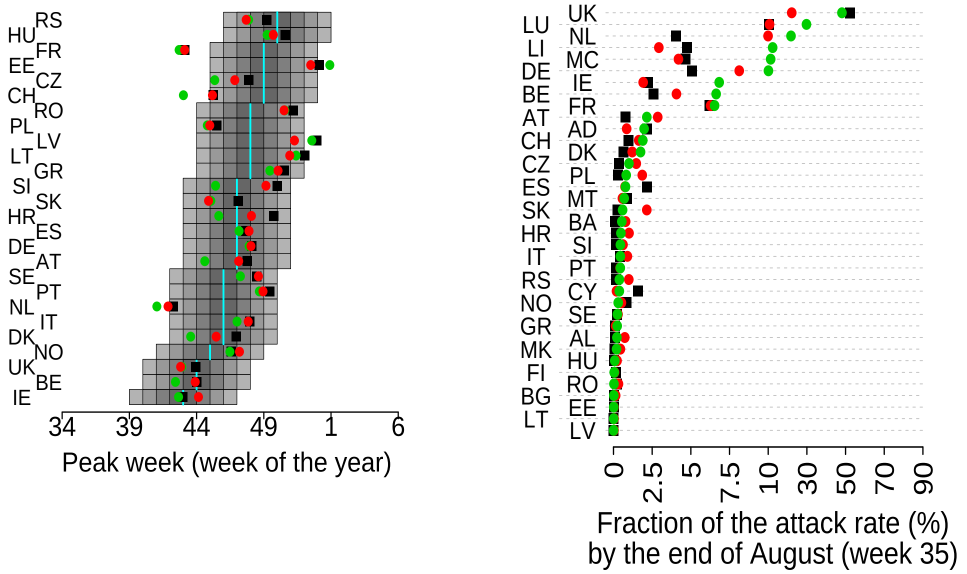

Figure S15: **a** Observed peak week (vertical cyan bars) for European countries covered by the WHO/Europe weekly influenza surveillance system plotted versus peak week as predicted in the baseline simulations (black squares), as predicted by assuming that cases are imported proportionally to the number of travellers from US and Mexico (green circles), as predicted by assuming that cases are imported proportionally to the population size (red circles) ; only the Autumn wave is considered for UK. **b** Activity up to week 35 in the three scenarios considered (colours as in **a**).

on the contrary, the epidemics in other countries, especially in Southern and Eastern part of Europe, strongly depend on importation of cases from local outbreaks ongoing in other European countries. As a consequence, predictions of the autumn peak do not differ much from that obtained in the baseline scenario shown in the main text in Spain, UK, Germany, the Netherlands, France while a delay is observed in other countries (e.g. Italy, Greece), even more remarkable if we do not consider cross-border diffusion (the mean deviation between observed and predicted peek week is 5 weeks on average).

4.3.2 Importation of cases from US and Mexico

In the baseline simulations we use a model for importing cases in the different European countries during the initial phase of the epidemic based on the analysis of travel–related cases in the early phase of the epidemic (see Sec. 1.2). Simulated imported cases are shown in Fig S3; they are quite consistent with observed data. In a variant of the model, we assumed that $p_c T$ in (1) would represent the number of daily travellers from US Mexico to the European country c as resulting from the analysis of airline data (see Fig. S4). The model would have failed to correctly predict the number of imported cases in Spain, as the model tends to reproduce better the importation of cases in countries with strong connections

with US (because of the much larger connection US-Europe with respect to Mexico-Europe). Moreover, the model would have overestimated the number of imported cases in Germany and the Netherlands, probably because flights to the airport hubs of Amsterdam, Frankfurt and Munich do not necessarily represent the final destination of passengers. In general, the simulated epidemics are much faster in those countries during the summer (see Fig. S15).

The number of passengers travelling from the different European countries to US and Mexico is not proportional to the population. For historical, cultural and, last but not least, economical reasons, the connections with US and Mexico are very heterogeneous. To what extent heterogeneity in the connection with US and Mexico affected the epidemic timing in Europe? To answer this question we assume that the number of cases imported from US and Mexico in the different European countries is proportional to the population of the countries (i.e., we do not change the overall number of imported cases, we only change the final destination). In this variant of the model, we assumed that constant p_c in (1) is proportional to the population size. This model would have completely failed to correctly predict the number of imported cases in the different countries (see Fig. S5). As resulting from Fig. S15, it should be noted that the probability of observing a summer wave in UK do not change much with respect to the baseline simulations shown in the main text. Moreover, we observed much faster epidemics in large countries less connected with US and Mexico (especially in the Eastern part of Europe). These results show that heterogeneity in the patterns of travels towards US and Mexico played a relevant role in determining the timing of the epidemic in Europe.

4.4 School closure

4.4.1 School closure in Europe

To show the effects of school closure in Europe we performed three experiments where we neglect heterogeneity in terms of school calendar, by assuming:

- Scenario 1) no summer/autumn holidays
- Scenario 2) the school calendar of UK (actually England and Wales) in all European countries (summer holidays start on 20 July and end on 7 September; autumn holidays start on 26 October and end on 30 October).
- Scenario 3) the school calendar of Finland in all European countries (summer holidays start on 30 May and end on 15 August; autumn holidays start on 29 October and end on 1 November);

Results are shown in Fig. 5 in the main text. By assuming no school holidays, it emerges that a relevant summer wave would have been observed in almost all European countries and the peak activity would have anticipated by months (the mean deviation between observed and predicted peek week is 11.4 weeks on average). By assuming the school calendar of Finland in all European countries, it emerges that no country would have experienced any summer wave. Moreover, a slight delay in the autumn peak week would have been observed (especially in

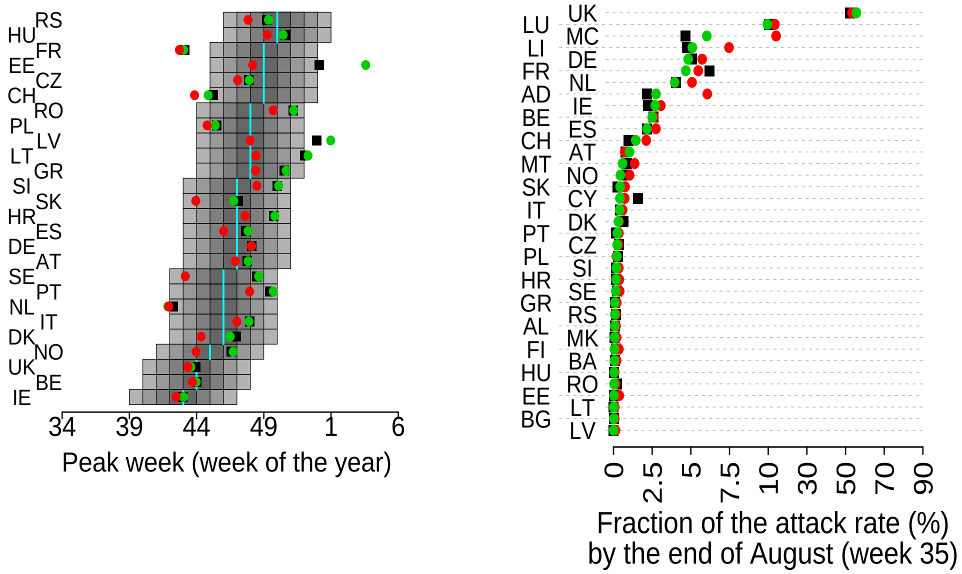

Figure S16: **a** Observed peak week (vertical cyan bars) for European countries covered by the WHO/Europe weekly influenza surveillance system plotted versus peak week as predicted in the baseline simulations (black squares), as predicted by assuming that schools in US do not reopen after the summer break (green circles), as predicted by assuming no summer holidays in US (red circles) ; only the Autumn wave is considered for UK. **b** Activity up to week 35 in the three scenarios considered (colours as in **a**).

countries less connected with US and Mexico) due to the lack of a well established epidemic in UK nor in other countries in the Western part of Europe during the summer (the mean deviation between observed and predicted peek week is 3 weeks on average). The opposite pattern is observed by assuming the school calendar of UK in all European countries. A summer wave would have been likely in the great majority of countries (especially in the Western part of Europe) and an anticipation of the autumn peak week would have been observed, due to epidemics ongoing in many Western countries and triggering the start of epidemics in the Eastern part of Europe (the mean deviation between observed and predicted peek week is 2.6 weeks on average). These results highlight the relevant role of school holidays in determining the epidemic timing.

4.4.2 School closure in United States

US experienced two epidemic waves, the first one in summer and the second one in autumn, probably because of summer holidays. To what extent the summer holidays in US affected the epidemic timing in Europe? To answer this question here we assume that schools do not reopen after the summer break. Results are shown in Fig. S16. It emerges that predictions of the autumn peak are not substantially different from that obtained in the baseline simulations shown in the main text (Spearman's correlation coefficient between peek week as predicted in

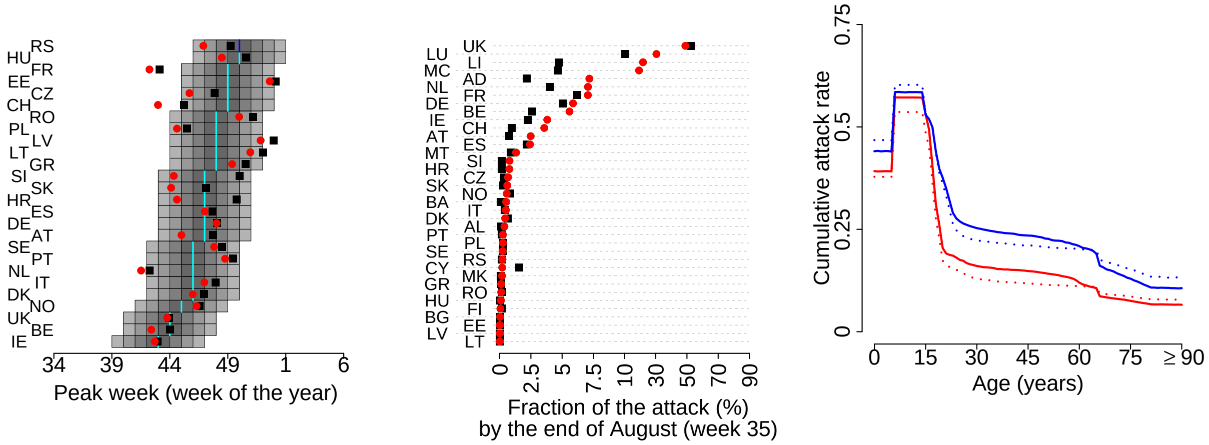

Figure S17: **a** Observed peak week (vertical cyan bars) for European countries covered by the WHO/Europe weekly influenza surveillance system plotted versus peak week as predicted in the baseline simulations (black squares), as predicted by assuming no transmission in workplaces (red circles); only the Autumn wave is considered for UK. **b** Activity up to week 35 in the two scenarios considered (colours as in **a**). **c** Cumulative attack rate by age in Ireland (blue lines) and Germany (red lines) in the baseline simulations (solid lines) and by assuming no transmission in workplaces (dashed lines).

the baseline simulations and by assuming that schools in US do not reopen after the summer break is 0.98, $p < 0.001$, mean deviation between peek weeks as predicted by the two models is 0.3 weeks on average). These results show that the timing of the epidemic in Europe was greatly determined by the timing of importation of cases during the early summer (i.e. the first wave in US). We also performed another secondary analysis where we assume no summer holidays in US. While it is very unrealistic to assume that importation of cases was not affected from summer holidays in US, these simulations allow to get insight into the possible effects of importation of cases from other countries during the summer. A very slight anticipation of the autumn peak week is observed and in general predictions improve in the Easter part of Europe (the mean deviation between observed and predicted peek week is 1.7 weeks on average), due to the continuous importation of cases during the summer. These results suggest that better predictions (in the Eastern part of Europe, e.g. Baltic countries) could have been obtained by considering connections with Eastern neighbouring countries (for instance Ukraine and Russia) where a sizeable epidemic was observed around the same period.

4.5 Workplaces

According to available data on the proportion of transmission in different social contexts for seasonal influenza (Cauchemez et al., 2008), the transmission model is parametrized so that in UK 18% of transmission occurs through contacts made at school, 30% within the households, 19% in workplaces and 33% in the general community. As stated above, we

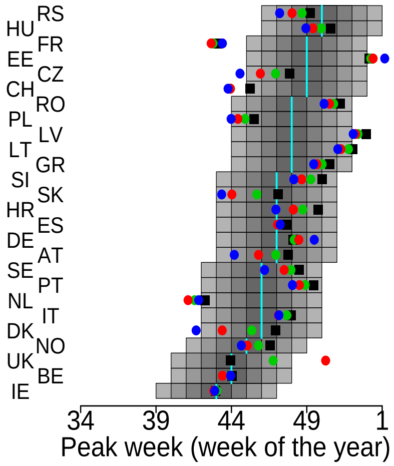

Figure S18: Observed peak week (vertical cyan bars) for European countries covered by the WHO/Europe weekly influenza surveillance system plotted versus peak week as predicted in the baseline simulations (black squares), as predicted by assuming that only 50% of cases are detected in the initial phase of the epidemic (blue circles), as predicted by assuming that only 20% of cases are detected in the initial phase of the epidemic (red circles), as predicted by assuming that only 10% of cases are detected in the initial phase of the epidemic (green circles) ; only the Autumn wave is considered for UK.

assume age-dependent susceptibility to infection. Specifically, in the baseline simulations we assume susceptibility to infection of children relative to adults equal to 2 (Fraser et al., 2009; Cauchemez et al., 2009). As a consequence of the age-dependent susceptibility to infection, the figure becomes 36% of cases in schools, 31% in households, 9% in workplaces and 24% in the general community.

Here we show the results as obtained by excluding transmission in the workplaces and thus considering a model in which the fraction of cases in the different social contexts is 33% of cases in schools, 30% in households and 37% in the general community. Results are shown in Fig. S17. It should be noted that the epidemic spreads slightly faster in both summer and autumn as a consequence of the larger number of cases generated by younger individuals in the general community. However, no remarkable differences are observed in the cumulative attack rate by age.

4.6 Case detection

According to the WHO daily reports (World Health Organization, 2009), 46 laboratory confirmed cases were reported in European countries from 27 April 2007 to 3 May 2009 (1 in Austria, 1 in Denmark, 2 in France, 6 in Germany, 1 in Ireland, 1 in Netherlands, 13 in Spain, 1 in Switzerland and 15 in UK). We initialized the simulated epidemics through the stochastic SEIR model describing the spread of the epidemic in Mexico and US in such a way that 46 imported cases were generated until 3 May 2009. Here we assume that the number of

laboratory confirmed cases is just a fraction of all imported cases, ranging from 10% to 100%. Fig. S18 shows that the prediction of the autumn peak is not very sensible to the percentage of detected cases up to 3 May 2009, as long as this is larger than 20%. This supports the robustness of the adopted method for setting the time in the simulations. The mean deviation between observed and predicted peek week increases from 2.2 to 3 weeks by decreasing the percentage of detected cases from 100% to 20%.

References

Ajelli, M. and Merler, S. (2008). The Impact of the Unstructured Contacts Component in Influenza Pandemic Modeling. *PLoS ONE*, 3(1):e1519.

Anderson, R. and May, R. M. (1992). *Infectious diseases of humans: dynamics and control*. Oxford, UK: Oxford University Press.

Anderson-Sköld, Y., Simpson, D., and Ødegaard, V. (2008). Humidity parameters from temperature: test of a simple methodology for European conditions. *Int. J. Climatol.*, 28:961–962.

Balk, D. and Yetman, G. (2004). The global distribution of population: evaluating the gains in resolution refinement. Technical report, Center for International Earth Science Information Network.

Belgian working group on influenza A(H1N1)v (2009). Influenza a(h1n1)v virus infections in belgium, may-june 2009. *Euro Surveill*, 14(28).

Cauchemez, S., Donnelly, C. A., Reed, C., Ghani, A. C., Fraser, C., Kent, C. K., Finelli, L., and Ferguson, N. M. (2009). Household transmission of 2009 pandemic influenza A (H1N1) virus in the United States. *N Engl J Med*, 361(27):2619–2627.

Cauchemez, S., Valleron, A.-J., Boëlle, P.-Y., Flahault, A., and Ferguson, N. M. (2008). Estimating the impact of school closure on influenza transmission from Sentinel data. *Nature*, 452(7188):750–754.

Chao, D. L., Halloran, M. E., Obenchain, V. J., and Longini, I. M. (2010). FluTE, a Publicly Available Stochastic Influenza Epidemic Simulation Model. *PLoS Comput Biol*, 6(1):e1000656.

Chowell, G., Ammon, C. E., Hengartner, N. W., and Hyman, J. M. (2006). Transmission dynamics of the great influenza pandemic of 1918 in Geneva, Switzerland: Assessing the effects of hypothetical interventions. *J Theor Biol*, 241(2):193–204.

Ciofi degli Atti, M. L., Merler, S., Rizzo, C., Ajelli, M., Massari, M., Manfredi, P., Furlanello, C., Scalia Tomba, G., and Iannelli, M. (2008). Mitigation measures for pandemic influenza in Italy: an individual based model considering different scenarios. *PLoS ONE*, 3(3):e1790.

Cowling, B. J., Fang, V. J., Riley, S., Peiris, J. S. M., and Leung, G. M. (2009). Estimation of the serial interval of influenza. *Epidemiology*, 20(3):344–347.

Diekmann, O., Heesterbeek, J. A., and Metz, J. A. (1990). On the definition and the computation of the basic reproduction ratio R0 in models for infectious diseases in heterogeneous populations. *J Math Biol*, 28(4):365–382.

Donnelly, C., Finelli, L., Cauchemez, S., Olsen, S., Doshi, S., Jackson, M., Kennedy, E., Kamimoto, L., Marchbanks, T., Morgan, O., Patel, M., Swerdlow, D., and Ferguson, N. (2011). Serial Intervals and the Temporal Distribution of Secondary Infections within Households of 2009 Pandemic Influenza A (H1N1): Implications for Influenza Control Recommendations. *Clinical Infectious Diseases*, 52(suppl 1):S123.

European Centre for Disease Prevention (2009). ECDC Situation Report, Influenza A(H1N1) infection, Update 3 June 2009.

European Centre for Disease Prevention and Control (2009). Preliminary report on case-based analysis of influenza A(H1N1) in EU and EEA/EFTA countries.

European Commission (2005). Key Data on Education in Europe 2005.

European Commission (2008). Organization of school time in Europe. Primary and general secondary education. 2008/09 school year.

Eurostat (2009). Statistics in focus. EU residents spent €94.2bn outside the EU for travel purposes in 2007.

Ferguson, N. (2007). Capturing human behaviour. *Nature*, 446(7137):733.

Ferguson, N. M., Cummings, D. A. T., Cauchemez, S., Fraser, C., Riley, S., Meeyai, A., Iamsirithaworn, S., and Burke, D. S. (2005). Strategies for containing an emerging influenza pandemic in Southeast Asia. *Nature*, 437(7056):209–214.

Ferguson, N. M., Cummings, D. A. T., Fraser, C., Cajka, J. C., Cooley, P. C., and Burke, D. S. (2006). Strategies for mitigating an influenza pandemic. *Nature*, 442(7101):448–452.

Fraser, C., Donnelly, C. A., Cauchemez, S., Hanage, W. P., Kerkhove, M. D. V., Hollingsworth, T. D., Griffin, J., Baggaley, R. F., Jenkins, H. E., Lyons, E. J., Jombart, T., Hinsley, W. R., Grassly, N. C., Balloux, F., Ghani, A. C., Ferguson, N. M., Rambaut, A., Pybus, O. G., Lopez-Gatell, H., Alpuche-Aranda, C. M., Chapela, I. B., Zavala, E. P., Guevara, D. M. E., Checchi, F., Garcia, E., Hugonnet, S., Roth, C., and Collaboration, W. H. O. R. P. A. (2009). Pandemic potential of a strain of influenza A(H1N1): early findings. *Science*, 324(5934):1557–1561.

Germann, T. C., Kadau, K., Longini, I. M., and Macken, C. A. (2006). Mitigation strategies for pandemic influenza in the United States. *Proc Natl Acad Sci U S A*, 103(15):5935–5940.

Ghani, A. C., Baguelin, M., Griffin, J., Flasche, S., Pebody, R., van Hoek, A. J., Cauchemez, S., Hall, I. M., Donnelly, C., Robertson, C., White, M. T., Barrass, I., Fraser, C., Bermingham, A., Truscott, J., Ellis, J., Jenkins, H., Kafatos, G., Garske, T., Harris, R., McMenamin, J., Hawkins, C., Phin, N., Charlett, A., Zambon, M., Edmonds, W. J., Catchpole, M., Leach, S., White, P., Ferguson, N. M., and Cooper, B. (2009). The Early Transmission Dynamics of H1N1pdm Influenza in the United Kingdom. *PLoS Curr Influenza*, page RRN1130.

Gojovic, M. Z., Sander, B., Fisman, D., Krahn, M. D., and Bauch, C. T. (2009). Modelling mitigation strategies for pandemic (H1N1) 2009. *Can. Med. Assoc. J.*, 181(10):673–680.

Goldstein, E., Paur, K., Fraser, C., Kenah, E., Wallinga, J., and Lipsitch, M. (2009). Reproductive numbers, epidemic spread and control in a community of households. *Math Biosci*, 221(1):11–25.

González, M. C., Hidalgo, C. A., and Barabási, A.-L. (2008). Understanding individual human mobility patterns. *Nature*, 453(7196):779–782.

Hahn, S., Donker, T., Meijer, A., Timen, A., van Steenbergen, J., Osterhaus, A., van der Sande, M., Koopmans, M., Wallinga, J., Coutinho, R., and Team, D. N. I. A. I. (2009). Epidemiology and control of influenza a(h1n1)v in the netherlands: the first 115 cases. *Euro Surveill*, 14(27).

Halloran, M. E., Ferguson, N. M., Eubank, S., Longini, I. M., Cummings, D. A. T., Lewis, B., Xu, S., Fraser, C., Vullikanti, A., Germann, T. C., Wagener, D., Beckman, R., Kadau, K., Barrett, C., Macken, C. A., Burke, D. S., and Cooley, P. (2008). Modeling targeted layered containment of an influenza pandemic in the United States. *Proc Natl Acad Sci U S A*, 105(12):4639–4644.

Haylock, M., Hofstra, N., Klein Tank, A., Klok, E., Jones, P., and M., N. (2008). A European daily highresolution gridded data set of surface temperature and precipitation for 1950-2006. *J. Geophys. Res.*, 113.

Hens, N., Ayele, G. M., Goeyvaerts, N., Aerts, M., Mossong, J., Edmunds, J. W., and Beutels, P. (2009). Estimating the impact of school closure on social mixing behaviour and the transmission of close contact infections in eight European countries. *BMC Infect Dis*, 9:187.

Lipsitch, M. and Viboud, C. (2009). Influenza seasonality: lifting the fog. *Proc Natl Acad Sci U S A*, 106(10):3645–3646.

Longini, I. M., Halloran, M. E., Nizam, A., and Yang, Y. (2004). Containing pandemic influenza with antiviral agents. *Am J Epidemiol*, 159(7):623–633.

Longini, I. M., Nizam, A., Xu, S., Ungchusak, K., Hanshaoworakul, W., Cummings, D. A. T., and Halloran, M. E. (2005). Containing pandemic influenza at the source. *Science*, 309(5737):1083–1087.

Merler, S. and Ajelli, M. (2010). The role of population heterogeneity and human mobility in the spread of pandemic influenza. *Proc Biol Sci*, 277(1681):557–565.

Merler, S., Poletti, P., Ajelli, M., Caprile, B., and Manfredi, P. (2008). Coinfection can trigger multiple pandemic waves. *J Theor Biol*, 254(2):499–507.

Miller, E., Hoschler, K., Hardelid, P., Stanford, E., Andrews, N., and Zambon, M. (2010). Incidence of 2009 pandemic influenza A H1N1 infection in England: a cross-sectional serological study. *Lancet*.

Novel influenza A(H1N1) investigation team (2009). Description of the early stage of pandemic (h1n1) 2009 in germany, 27 april-16 june 2009. *Euro Surveill*, 14(31).

Poletti, P., Caprile, B., Ajelli, M., Pugliese, A., and Merler, S. (2009). Spontaneous behavioural changes in response to epidemics. *J Theor Biol*, 260(1):31–40.

Rizzo, C., Declich, S., Bella, A., Caporali, M. G., Lana, S., Pompa, M. G., Vellucci, L., and Salmaso, S. (2009). Enhanced epidemiological surveillance of influenza A(H1N1)v in Italy. *Euro Surveill*, 14(27).

Shaman, J. and Kohn, M. (2009). Absolute humidity modulates influenza survival, transmission, and seasonality. *Proc Natl Acad Sci U S A*, 106(9):3243–3248.

Shaman, J., Pitzer, V. E., Viboud, C., Grenfell, B. T., and Lipsitch, M. (2010). Absolute humidity and the seasonal onset of influenza in the continental United States. *PLoS Biol*, 8(2):e1000316.

White, L. F., Wallinga, J., Finelli, L., Reed, C., Riley, S., Lipsitch, M., and Pagano, M. (2009). Estimation of the reproductive number and the serial interval in early phase of the 2009 influenza a/h1n1 pandemic in the usa. *Influenza Other Respi Viruses*, 3(6):267–276.

World Health Organization (2009). Situation updates - Pandemic (H1N1) 2009. `http://www.who.int/csr/disease/swineflu/updates/en/index.html`.

Xing, Z. and Cardona, C. J. (2009). Preexisting immunity to pandemic (H1N1) 2009. *Emerg Infect Dis*, 15(11):1847–1849.

Yang, Y., Sugimoto, J. D., Halloran, M. E., Basta, N. E., Chao, D. L., Matrajt, L., Potter, G., Kenah, E., and Longini, I. M. (2009). The transmissibility and control of pandemic influenza A (H1N1) virus. *Science*, 326(5953):729–733.

Zimmer, S. M. and Burke, D. S. (2009). Historical perspective–Emergence of influenza A (H1N1) viruses. *N Engl J Med*, 361(3):279–285.
